# Diurnal Variation in Biomarkers of Exposure to Endocrine-Disrupting Chemicals and Their Association with Oxidative Damage in Norwegian Adults: The EuroMix Study

**DOI:** 10.3390/toxics10040181

**Published:** 2022-04-07

**Authors:** Samuel Olushola Abimbola, Christina Xeni, Amrit Kaur Sakhi, Behzad Heibati, Trine Husøy, Hubert Dirven, Konstantinos C. Makris

**Affiliations:** 1 Cyprus International Institute for Environmental and Public Health, Cyprus University of Technology, Limassol 3041, Cyprus; sa.abimbola@edu.cut.ac.cy (S.O.A.); christina.xeni@cut.ac.cy (C.X.); behzad.heibati@cut.ac.cy (B.H.); 2 Norwegian Institute of Public Health (NIPH), P.O. Box 222 Skøyen, N-0213 Oslo, Norway; amritkaur.sakhi@fhi.no (A.K.S.); trine.husoy@fhi.no (T.H.); hubert.dirven@fhi.no (H.D.); 3 Center for Environmental and Respiratory Health Research (CERH), Faculty of Medicine, University of Oulu, Biocenter, FI-90014 Oulu, Finland

**Keywords:** endocrine-disrupting chemicals (EDCs), circadian rhythm, diurnal variation, phthalates, bisphenols, parabens, personal care products

## Abstract

Much evidence on the adverse health effects of endocrine-disrupting chemicals (EDCs) has accumulated during recent decades. EDCs are commonly found in various foods and personal care products (PCP). Data documenting a diurnally varying EDC metabolism in humans is scarce. This study examined (i) the time-of-day effect on the diurnal magnitude and variance of urinary biomarkers of exposure to EDCs, and (ii) the association between EDC exposures and oxidative damage in a Norwegian adult subpopulation. This was a cross-sectional panel study using biobanked samples from the EuroMix project. During a typical weekday, participants were asked to collect all day’s urine voids and record dietary and PCP habitual uses in a diary. Collected time stamps of urine voids were classified into three distinct periods in the day (morning 6 a.m.–12 p.m., mid-day 12 p.m.–6 p.m., evening 6 p.m.–6 a.m.). Questionnaires regarding demographic characteristics, personal care product usage, and dietary habits were completed. Urinary levels of EDCs (phthalates, parabens, and bisphenols) were measured using mass spectrometry and adjusted for urinary volume using specific gravity. Urinary 4-hydroxynonenal (4HNE), a lipid peroxidation marker, was measured using an immunoassay kit. Linear mixed-effect models identified EDCs under the influence of a diurnal variation effect that was adjusted for dietary habits and PCP use and examined associations between EDC and 4HNE. *p*-values were FDR-adjusted. Most phthalates appeared to be diurnally varying with higher urinary levels towards the evening (*q* < 0.001) than those measured during mid-day; this strong diurnal variation effect was not present for parabens and bisphenols. Significant (*q* < 0.001) positive associations were observed between all phthalates, parabens, and bisphenols (except bisphenol S) and 4HNE. This study’s findings highlighted the diurnal variation of excretion for certain EDC, but not for others, in real-life conditions. The degree of EDC chronotoxicity in distinct diurnal windows of the day warrants further investigation with longitudinal human studies.

## 1. Introduction

Much scientific interest and discussion about the impact of endocrine-disrupting chemicals (EDCs) on humans and the environment have emerged during recent decades [1]. According to the Organization for Economic Co-operation and Development (OECD), “An endocrine disruptor is an exogenous substance or mixture that alters function(s) of the endocrine system and consequently causes adverse health effects in an intact organism, or its progeny, or (sub)populations” [2]. The economic costs of diseases and dysfunction associated with exposure to EDCs in the European Union (EU) have been estimated to be hundreds of billions of euros per year [3].

EDCs are ubiquitous in most environmental media, while humans may come in contact with them via all routes and various sources of exposure. Prominent examples of EDC classes are phthalates, bisphenols, and parabens, which hold specific characteristics of non-persistent chemicals, such as limited lipophilicity and relatively short half-lives of elimination [4,5]; indeed, the biological half-life of metabolites of phthalate/DINCH in urine was <12 h for all measured compounds [6,7,8]. BPA has a half-life of <6–7 h [9,10], while the half-lives of most parabens range between 13 h and 29 h [7,11].

Phthalates have been widely used as plasticizers, providing flexibility to rigid plastics. They are used in food-contact materials, toys, as emulsifying agents and solvents in cosmetics, and excipients in pharmaceuticals [12,13]. Phthalates have been associated with a series of non-communicable diseases, ranging from reproductive toxicity in both adults and adolescents across genders, impaired infant reproductive health, insulin resistance and type II diabetes, hormonal cancer, etc. [4,14,15,16]. Parabens are widely used as preservatives in cosmetics and pharmaceutical products [17]. Bisphenols are used primarily in the production of polycarbonate plastics and epoxy resins [18]. Parabens and bisphenols have been associated with respiratory diseases, atopic dermatitis, allergic rhinitis, reproductive dysfunction, or cancer [4,19,20,21,22].

The widespread usage of phthalates, parabens, and bisphenols leads to ubiquitous, constant, and potentially inevitable exposure in humans [4,23]. Due to the short half-life of the aforementioned EDCs, variations in EDCs’ body burden may depend on the source of EDC exposure and the time of exposure [5,8,24]. Therefore, the time of the sampling is important for detecting within- and between-day variations in human exposure. Interestingly, temporal within- and between-day variations in concentrations have been shown for metabolites of phthalates, bisphenols, and parabens [5,8,9,11,23,24,25,26]. One of these studies examined the impact of time-of-day expression of enzymatic/metabolic activity (CYP2E1) on the observed diurnal (within-day) variability of exposure to disinfection byproducts [24]. Non-persistent EDCs may be characterized by low intraclass correlation coefficient (ICC) estimates (<0.5), where single-spot urine data would likely lead to a higher probability of exposure misclassification [5,8,23]. Time-resolved, repeated measures in human studies would be needed to reliably estimate the magnitude and variance of exposure to non-persistent EDC [5,23].

Synchronization between the central circadian clock and the peripheral liver clock’s enzymatic activity may be key in controlling the hepatic enzyme-driven EDC metabolism patterns [24,27]. The interplay between EDC exposure patterns and their Phase I/II metabolism rates as influenced by the endogenous clock-dictated CYP450 enzymatic activity patterns remains much unexplored. These EDCs have been shown to induce oxidative stress in human studies, initiating lipid peroxidation in cellular membranes [28]. Lipid peroxidation may generate reactive-oxygen-species-induced tissue injury via the formation of lipid peroxidation products, such as 4HNE [29]. 4HNE has been involved in various pathologies, such as metabolic diseases, neurodegenerative diseases [30], cancers [31], alcoholic liver disease [32], and chronic obstructive pulmonary disease [33]. 

The biological hypothesis that the liver-clock-driven metabolism of EDC diurnally differs by chemical class-specific enzyme systems was tested in a repeated-measures adult study in Norway. The lipid peroxidation potential of these EDC exposures associated with dietary and personal-care-product use (PCP) was also considered. Thus, the main objectives of this study were to (i) investigate the influence of the time-of-day effects on the magnitude and variation of biomarkers of exposure to EDCs in Norwegian adults and (ii) evaluate the association between the biomarkers of exposure to EDCs with the biomarkers of lipid peroxidation (4-hydroxynonenal, 4HNE) measurements.

## 2. Materials and Methods

### 2.1. Data Collection

**Figure 1:**
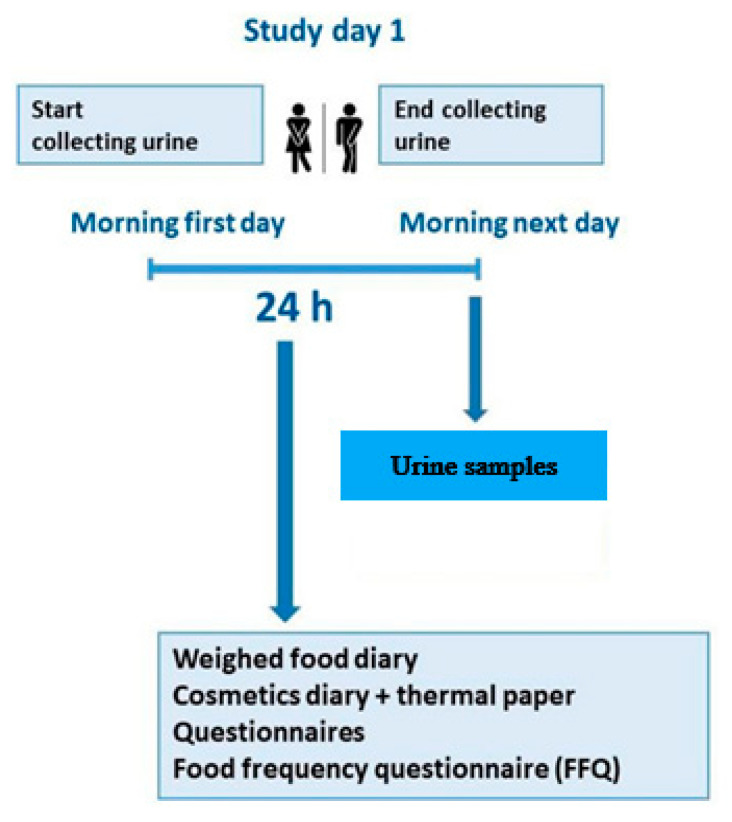
Graphical presentation of the human biomonitoring study design.

A detailed description of the EuroMix study, including the study’s inclusion/exclusion criteria, can be found in Husøy et al. [34]. In short, a total of 144 participants (44 men and 100 women) were recruited amongst employees of governmental institutes and authorities and universities in the counties of Oslo and Akershus in Norway between September 2016 and November 2017. The recruited participants might not be a representative sample of the overall Norwegian population, but this was not the aim of the biomonitoring study. A sample size of approximately 150 was considered sufficient as previous studies conducted by our team using comparable sample collection and study design had similar sample sizes [34,35,36]. Participants collected every urination during a 24-h period in separate containers and marked them with the time and date. The day after collection, the samples were measured and stored as pools of urine combining all the voids into three time periods/pools (morning time group 1 = 06:00–12:00, mid-day time group 2 = 12:00–18:00, evening time group 3 = 18:00–06:00 the next day) (Figure 1). Of the 144 participants, six did not submit urine for one of the time periods; these were excluded from the analyses. 

A total of 144 participants (44 males aged 25–72 years and 100 females aged 24–72 years) completed a diary of their weighed food record, a cosmetic diary, a food frequency questionnaire (FFQ) (for the 24 h period), and a questionnaire on socio-demographic and lifestyle characteristics (Figure 1). The study was approved by the Regional Committees for Medical and Health Research Ethics (REK ID no 2015/1868) on the 19th of November, 2015, and all the participants provided their written informed consent.

### 2.2. Biomarkers

Data on biomarkers of exposure to EDCs based on the 3 urine time group pools were included in this analysis. Twelve phthalate metabolites (MEP, MiBP, MnBP, MBzP, MEHP, MEHHP, MEOHP, MECPP, oh-MiNP, oxo-MiNP, cx-MiNP, oh-MPHP) and two metabolites of DINCH (oh-MINCH and oxo-MINCH]) were determined in the three urinary time pools of the day, using liquid chromatography coupled to mass spectrometry (LC-MS-MS) as described in Sabaredzovic et al. [37]. These metabolites were from eight parent compounds, as shown in Table S1. The concentrations of DEHP, DiNP, and DINCH in urine were given as an estimated sum of their metabolites (total ug), after adjusting for molecular weights, as sumDEHP (MEHP, MEHHP, MEOHP, MECPP, MMCHP), sumDiNP (oh-MiNP, oxo-MiNP, cx-MiNP), and sumDINCH (oh-MINCH, oxo-MINCH). Four parabens (MEPA, ETPA, PRPA, and BUPA) and three environmental phenols (BPA, BPS, and BPF) were also included (units ug/L) and were determined using the LC-MS-MS method [38]. Quality control and quality assurance details of the measured biomarkers can be found in the original study by Husøy et al. [34]. Urinary 4-hydroxynonenal (4-HNE, ug/L) was determined using immunoassay following the manufacturer’s instructions (Wuhan Fine Biotech Co., Ltd., China). The inter- and intra-coefficients of variability for the 4-HNE assay were 3–4% and 5–7%, respectively.

### 2.3. Data Handling

#### 2.3.1. Data Cleaning

The following datasets were obtained, including their timestamps in the day: Demographics, 24 h food diaries, 24 h PCP diaries, EDC, and 4HNE measurements. Although data on thermal paper handling was collected, it was not included in this analysis as the number of participants who reported handling thermal paper was small (24%). In order to carry out the analyses, the datasets of the food and PCP diaries were modified. The times recording each meal and PCP use were grouped into a three-level categorical variable to match the time groups for urine collection: Time group 1 (morning, 06:00–12:00), time group 2 (mid-day 12:00–18:00), and time group 3 (evening 18:00–06:00). Time group 2 (mid-day) was set as the reference level.

The 44 different food categories recorded in the study were reclassified into 15 group categories, as follows: Beverages, sweets, bread, fish, grain, dairy, egg, cakes, fruit, vegetables, butter and oil, potatoes, meat, cheese, and others. Each food group consumed was transformed into a binary categorical variable with 0 = ‘did not eat’ and 1 = ‘eat’. All PCP products were categorized into the following 26 new classes: Shower gel, shampoo, conditioner, deodorant, facial cleanser, facial moisturizer, body lotion, anti-wrinkle cream, sunscreen, mouth wash, toothpaste, perfume, lip gloss/stick/balm, foundation, intimate soap, hand cream, foot cream, hair styling product, hair treatment, eye make-up product, rouge and powder product, make-up remover product, shaving product, antibacterial product, ‘oils’ product, and hand soap. Certain products that could not be classified into any of the categories due to the ambiguity of the product description in the dataset were not used in our analysis; these products were used by less than 5% of the participants: Vaseline, wax, serum, lenses, condom, nail polish, paint (sens) on hand/arms, contact lenses, waxing, concealer pencil, nasal spray, physiological saltwater, carnival make-up, and floss. Each PCP product was defined as a binary variable with 0 = ‘did not use it’ and 1 = ‘used it’.

#### 2.3.2. Dealing with Missing Values

We removed variables with > 80% of their values as missing values. As for the variables that had ≥20% but less than 80% of their values as missing values, we proceeded with imputation using Monte Carlo imputation [39].

#### 2.3.3. Data below Limit of Detection

Outcome measurements below the limit of detection (LOD) were substituted with LOD/2. Outcomes with >60% but <90% values below detection were used as binary variables (above LOD vs. below LOD). Outcomes with values >90% below LOD were not used in the final analysis.

#### 2.3.4. Data Adjustment

To correct for urinary dilution, specific gravity (SG) was measured in all urine samples. SG-adjusted concentrations were used in the statistical analyses. Unlike creatinine-adjusted values, SG is less affected by factors such as diet, season, age, muscle mass, and gender [40]. In essence, all measurement values for the biomarkers of exposure to EDCs and the biomarker of effect (4HNE) were SG-adjusted using the Formula (1):

CHEM_SG_Adj_ (µg/L) = CHEM_i_ ((SG_m_ − 1)/(SG_i_ − 1)) (1)

where CHEM_SG_Adj_ is the SG-standardized analyte concentration, CHEM_i_ is the observed analyte concentration, SG_i_ is the specific gravity of the urine sample, and SG_m_ is the median specific gravity for the study population.

### 2.4. Statistical Analysis

Descriptive statistics (percentiles, min, and max) were calculated for the biomarkers of exposure to EDCs and 4HNE. Boxplots were constructed to visualize the time-of-day effect on EDCs and 4HNE.

Linear mixed-effect (LME) models were constructed for each biomarker of exposure to EDCs (log-transformed and SG-adjusted) accounting for the repeated measures within subjects. Fixed-effect variables were the time groups (mid-day time group 2 was the reference) along with the covariates of sex, age, and BMI. Select food groups and PCP types (transformed and imputed as binary variables) earlier found to be associated with the EDC levels in the original project analysis [34] were also included in the regressions: (i) For phthalates, meat, bread, beverages, butter, oil and PCPs of shower gel, hand cream, toothpaste, antiwrinkle cream, shaving products; and (ii) for both parabens and bisphenols, meat, bread, beverages, butter and oil, and lip products (BPA only). Participant id was included in the LME as the random-effect variable. Smoking status, along with educational level, was not included in the model, as the sample population was considered highly educated, with nearly 80% of the participants having at least a university degree and >84% of the participants not being active smokers. A correlation coefficient matrix was constructed to observe correlations between the studied EDCs, including 4HNE. 

To explore the association between the biomarkers of exposure to EDCs and 4HNE, the second set of regression models was constructed using 4HNE (log-transformed and SG-adjusted) as the response variable. Each biomarker of exposure to EDCs was used as a fixed-effect variable, including covariates, such as sex, age, and BMI. The participants were considered random effects in the model. 

In both sets of models, multiple testing correction was performed using the Benjamini–Hochberg (false discovery rate, FDR) method considering all regression models. A *q*-value < 0.05 indicates the statistical significance of an association after controlling the FDR at 5%. All analyses were performed in R (v.4.0.5) with RStudio (1.4.1717). 

A sensitivity analysis was performed for the first set of regression models, using the frequency of consumption (in each time group of the day) for select food groups as a continuous function; associations and the regression coefficients remained essentially the same.

## 3. Results

### 3.1. Population Characteristics

The EuroMix study population (*n* = 144) had a mean age of 43 years with normal BMI, being mostly females, highly educated, with nearly 80% of the study participants having a university degree. There were no regular smokers, with 87% either having never smoked before or having quit smoking and the remaining 13% being active but only occasional smokers. The mean weights of the males and the females were 82 kg (standard deviation, SD = 8.5) and 65 kg (SD = 8.9), respectively (Table 1). Detailed descriptive information on diet from weighed food records and FFQ, as well as PCP use by the study population, was previously reported [34].

### 3.2. Biomarkers of Exposure/Effect

#### 3.2.1. Urinary Biomarkers of Exposure to Phenols, Phthalates, and Parabens

Only 4% of the urine samples had BPA levels <LOD, while 71% and 96% of the samples had levels < LOD for BPS and BPF, respectively. There were varying detection levels for the parabens; all urine samples had MEPA concentrations above the LOD, while 1%, 35%, and 50% of the samples were below LOD for ETPA, PRPA, and BUPA, respectively (Table S2). The non-quantified rates (samples below LOD) for the metabolites of phthalates/DINCH ranged between 0% and 24%; all values were above LOD for 10 phthalates—MeP, MiBP, MnBP, MBzP, MEHHP, MEOHP, MECPP, oh_MiNP, oxo_MiNP, and cx_MiNP. The <LOD percentages for MEHP, MMCHP, oh-MINCH, oxo-MINCH, and oh-MPHP were 12%, 3%, 11%, 4%, and 24%, respectively (Table S1).

Of all the EDC biomarkers, the highest concentrations were observed in MEPA (morning, 18519 ug/L), ETPA (night, 2038 ug/L), and MEP (mid-day, 1990 ug). More details on the percentiles of the urinary EDCs by the time of day and overall are presented in Table 2. The concentrations of all the metabolites of phthalates/DINCH in the urine samples followed a diurnal variation pattern with the lowest concentrations observed during mid-day, with levels being ordered by evening > morning > mid-day (Figure S2). Urinary concentrations were higher in males than females for all phthalates except for MEP (Figure S3). Urinary BPA levels were higher in the morning, with a median value of 1.39 ug/L (IQR:0.62, 2.44), than at night (1.17 ug/L; IQR:0.68, 1.97) (Table 2, Figure S2). The diurnal concentration pattern for BPS could not be clarified as the lower concentration quartiles were below the LOD (Table 2). Concentrations of BPA in males were slightly higher than in females (Figure S3). The diurnal profile for MEPA followed the order of evening (7.32 ug/L; IQR: 3.2, 27.7) > morning (6.31 ug/L; IQR: 3.4, 37.2) > mid-day (5.85 ug/L; IQR: 2.95, 24.3). The diurnal profile of urinary PRPA and ETPA followed the order of morning (0.25 ug/L; IQR: 0.02, 2.8) > night (0.17 ug/L; IQR: 0.03, 3.40) > mid-day (0.10 ug/L; IQR: 0.02, 1.16), and morning (1.24 ug/L; IQR: 0.52, 3.43) > mid-day (1.08 ug/L; IQR: 0.40,3.19) > night (0.84 ug/L; IQR: 0.34, 3.26), respectively. The concentrations of all parabens were higher in females than in males (Figure S3).

The magnitude of urinary 4HNE levels by time group in the 24 h period did not diurnally vary (*p* > 0.05), because of its anticipated slower rate of formation at the expense of the faster EDC metabolism. However, higher levels of 4HNE were observed among males than in females (Figure S3).

#### 3.2.2. Association between Time of the Day and the Biomarkers of EDC Exposure and Effect

**Figure 2:**
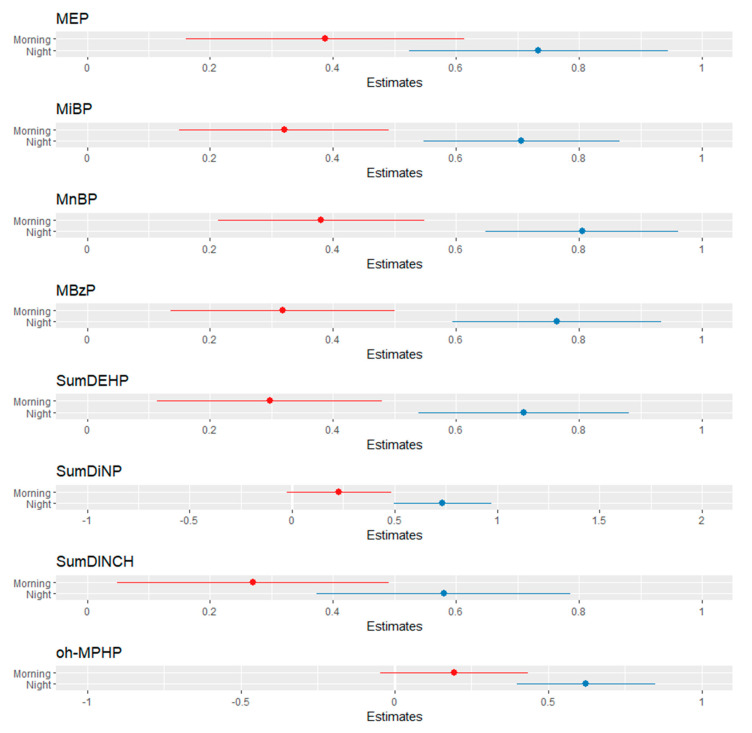
Forest plot visualization of the linear mixed-effect models of SG-adjusted metabolites of phthalates/DINCH as a function of time of urine sampling in the day adjusted for dietary habits and personal-care-product use (reference: mid-day = zero point).

The LME models for most phthalates/DINCH metabolites showed significant associations between urinary EDC concentrations and the time of the day (Figure 2, Table S4i–xiii). All models were adjusted for age, sex, BMI, dietary habits, and PCP use. Statistically significant positive associations were found between time (morning) and MiBP (β = 0.32, 95% CI: 0.15, 0.49, *q* < 0.001), MnBP (β = 0.38, 95% CI: 0.21, 0.55, *q* < 0.001), MBzP (β = 0.32, 95% CI: 0.14, 0.50, *q* = 0.006), MEP (β = 0.39, 95% CI: 0.16, 0.61, *q* = 0.006), and SumDEHP (β = 0.30, 95% CI: 0.11, 0.48, *q* = 0.011), but not with SumDiNP, SumDINCH, and oh-MPHP. Statistically significant associations (*q* < 0.001) were also found between time (night) and all phthalate metabolites (Table S4). There were statistically significant differences in the concentration of some phthalates (MiBP, MnBP, MBzP, SumDEHP, SumDiNP, and SumDINCH) by sex (Table S4). However, only MiBP, MnBP, SumDEHP, and SumDINCH remained significantly different (*q* < 0.05) after applying the Benjamini–Hochberg correction. The effect of time on the concentration of phthalates was unaffected by BMI (Table S4).

**Figure 3:**
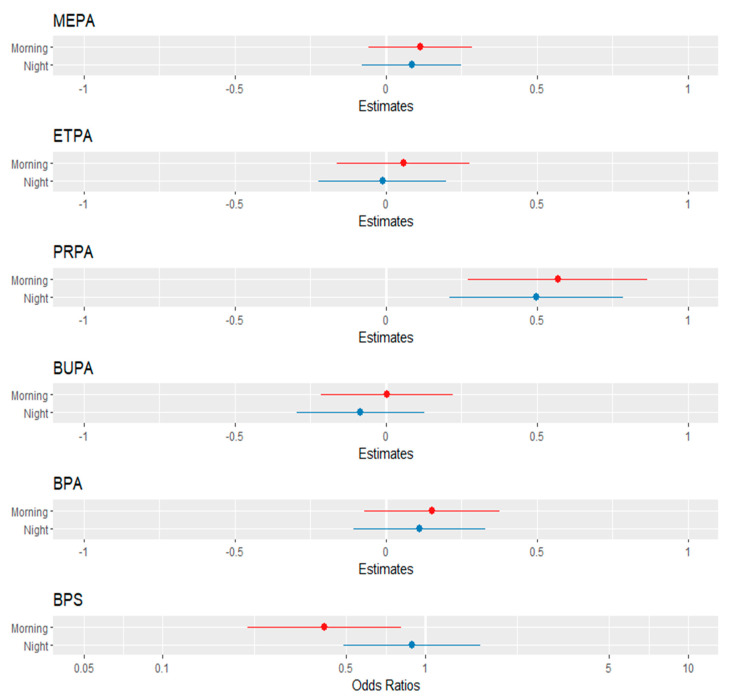
Forest plot visualization of the linear mixed-effect models of SG-adjusted parabens and bisphenols as a function of time of urine sampling in the day adjusted for dietary habits and personal-care-product use (reference: mid-day = zero point on *x*-axis).

In the case of parabens, a statistically significant positive association was only found for PRPA and time (morning) (β = 0.57, 95% CI: 0.27, 0.86, *q* < 0.001) as well as time (night) (β = 0.50, 95% CI: 0.21, 0.86, *q* = 0.006). For the rest of the parabens, including bisphenols, there were no statistically significant associations between the urinary concentration levels of MEPA, ETPA, BUPA, and BPA and time of urination groups of the day; BPS was significantly associated with time (morning) (β = 0.41, 95% CI: 0.21, 0.81, *q* = 0.047) but not with time (night) (Figure 3, Table S4). Statistically significant differences by sex were observed in the concentrations of PRPA and MEPA (*q* < 0.001 and *q* = 0.043, respectively). There was little differentiation in the 4HNE levels among the three time groups of the day (Table S4).

#### 3.2.3. Association between Biomarkers of Exposure to EDCs and the Biomarker of Oxidative Damage

An LME model was employed to test the hypothesis that the diurnally varying biomarkers of exposure would be associated with the biomarker of enzymatic activity/oxidative damage (4HNE). Statistically significant associations were found between 4HNE and all metabolites of phthalates/DINCH, phenols, and parabens, except for BPS. Significant associations remained for all biomarkers of exposure to EDCs after applying the Benjamini–Hochberg correction (*q* value < 0.001) (Table 3). The regression models on the associations between biomarkers of exposure to EDCs and the biomarker of effect (4HNE) can be found in Table S5.

Sex significantly impacted the association between 4HNE and the biomarkers of exposure to phthalates, phenols, and parabens; the magnitude of 4HNE associations was consistently higher in males for all EDCs tested (*q*-value < 0.001) (Table S5). Age and BMI had no impact on the association between the biomarkers of exposure and the biomarker of effect.

## 4. Discussion

We found that all urinary biomarkers of exposure to phthalates/DINCH followed a diurnal pattern, peaking in the evening and continuing to be high in the morning, and then gradually declining during the day with the lowest concentrations measured at mid-day. This consistent pattern was not so obvious for parabens or bisphenols. Earlier research by Gängler et al. [23] looked at the effect of the time of day on the variability of urinary biomarkers of exposure to disinfectant by-products. They observed that the levels of urinary trihalomethanes (THMs) during the day followed a diurnal pattern; higher levels of THM were measured in urine samples collected during the evening and lower levels in those collected during mid-day before and after engaging in household cleaning activities, respectively [23].

It was also speculated that the chrono-varying diurnal trend for phthalates could be associated with the variance in the day liver-clock-driven enzymatic expression of CYP450. Cytochromes P450 (CYPs) are a superfamily of enzymes that play a leading role in drug metabolism and detoxification, contributing to the metabolism of up to 75% of drugs. Biological pathways in which CYPs are involved, including drug and xenobiotic metabolism, have been shown to display circadian variation [41,42]. Several previous studies indicated that these CYPs are not directly regulated by the CLOCK/BMAL1 heterodimer, but indirectly through clock-regulated genes, such as PARbZip [43] and nuclear receptors [44]. While animal studies have shown circadian-based hepatotoxic effects of CYP450 activity as a result of xenobiotics treatment, only three human studies [23,45,46] have previously shown the influence of circadian/diurnal variation on the metabolism and possible chronotoxicity of environmental chemicals, including EDC. The diurnal variation in the magnitude of the EDC metabolites was evident in our study, but we are unsure whether this would be exclusively ascribed to the diurnal time-dependent expression of such liver CYPs enzyme systems. The possibility that food intake and its timestamp in the day may not have been sufficiently adjusted for remains, and it could be partially held responsible for some of the EDCs’ diurnal differences observed. It is also possible that the lack of considering the previous day’s food intake habits and quantities would have influenced the observed diurnal trends on the biomarkers of exposure to EDCs. Further, we did not take into account the EDC concentrations in the studied food items, which might lead to residual confounding. The sensitivity-based regression models using the number of food items consumed in each time group of the day did not alter the direction or strength of the diurnal time effect on the biomarkers of exposure to EDC after adjusting for the possible confounding effect of dietary intake. Select food items consumed in each time group of the day were chosen for inclusion in the regression models in two ways: (i) Added as a yes/no variable and (ii) added as a continuous variable in grams consumed per time group. In both cases, the food intake did not influence the effect size of the diurnal effects on urinary EDC. 

Interestingly, this diurnal variation in the concentration of EDC metabolites was not consistently observed for parabens and bisphenols. Except for paraben PRPA, the rest of the parabens (MEPA, ETPA, BUPA) and bisphenols (BPA and BPS) did not follow the same diurnal variation as seen in phthalates. A notable difference between the phthalates and the parabens is that, on average, the urinary half-lives of excretion for parabens are longer (ranging between 13 and 29 h) [5,9] when compared with those of phthalates (<12 h) [4,5,6]. This difference may be important for the monitoring of diurnal (24-h) variation effects on the metabolism of short-lived chemicals. Studies also suggest that parabens are metabolized by carboxylesterases (CES) [47,48,49], with carboxylesterase1 (CES1) being the predominant CES enzyme in the liver, responsible for the metabolism of short-chain parabens (e.g., ETPA, PRPA, BUPA, and MEPA), while carboxylesterase2 (CES2) is more prevalent in skin tissue [49]. As such, the route of exposure plays an important role in the metabolism of parabens, as the level of absorption following oral intake has been shown to be higher than the levels following dermal uptake [50]. Findings on the exposure pattern of the participants to parabens based on the exposure to food or PCPs that were significantly correlated with the observed concentrations have been previously reported [34]. Hepatic CES enzymes have been shown to exhibit circadian variation in animal studies with higher levels in the dark phase than in the light phase [42,51,52]. However, Zhang et al. [43] clearly illustrated that although variations followed this pattern of diurnal expression, fluctuations in the expression of CES transcripts were low, with levels of hepatic CES isoenzymes during the dark phase being only slightly higher than levels during the light phase with additional minor troughs during the dark phase. In addition, the inhibitory influence of parabens on CYPs enzyme systems is also well known. There is some evidence that parabens may have an inhibitory effect on CYP450 enzymatic activity [53], thereby altering the pharmacokinetics of co-occurring EDC, such as BPA [54]. However, this panel study was not designed to study the biological mechanism of the observed diurnal variation patterns in the EDC metabolism as a result of the corresponding hepatic enzyme systems for each EDC class. There is a need for further studies to establish the potential impact of co-exposure on the metabolic/pharmacokinetic fate of individual EDCs.

To the best of our knowledge, this seems to be the first human study to report significant associations between 4HNE and EDCs (bisphenols, phthalates, and parabens). Most of the EDCs in this study showed associations with the marker of lipid oxidative damage (4HNE). 4HNE is a well-known biomarker of oxidative stress-induced lipid peroxidation both in animal models [55,56] and in human studies [57], and it has been associated with the pathogenesis and onset of cancer [58], arthritis [59], cardiovascular diseases, and diabetes [60]. The association between EDCs and oxidative damage observed in this study is supported by findings in earlier studies [61]. Urinary BPA was positively associated with 8-hydroxy-2′-deoxyguanosine (8-OHdG), 8-isoprostane, and 4-hydroxy-2-nonenal-mercapturic acid (HNE-MA), while BPF was associated with HNE-MA and 8-isoprostane in a Chinese cross-sectional study [62,63]. Similar associations between bisphenols and oxidative damage have been found in pregnant women and fetuses/newborns [64,65], or children and adolescents [66,67]. DEHP exposure in adolescents affected lipid metabolism by regulating the expression of lipid metabolism-related genes [68]. Urinary parabens have also been associated with oxidative stress markers in human studies [28].

In addition to chemical exposures, the oxidative stress imbalance exceeding the antioxidant defense mechanisms may also be associated with a suite of lifestyle and behavioral risk factors, such as alcohol or smoking [69]. A positive association was found earlier between smoking/tobacco exposure [70,71,72] and alcohol [73,74] consumption with increased levels of oxidative damage (4HNE). Moreover, a significant association was found between BMI and oxidative damage, with studies reporting a positive effect of obesity on increased 4HNE [73,75]. However, the majority of our study population was composed of non-active smokers and non-obese, thus their lifestyle characteristics were anticipated to have little influence on the variation of the observed 4HNE levels.

4-HNE-associated oxidative stress may also be accompanied by changes in enzyme activity patterns. For example, elevated formation of 4HNE was associated with the expression of GSTA4–4, CYP2E1, and Hsp70 proteins in the mitochondria of PC12 cells [76]. Based on a study with CYP2E1-deficient mice, 4HNE was elevated in mice exposed to environmental chemicals when compared with the CYP2E1-knock out mice [77]. Disinfection by-product chemical toxicity was mediated by its free-radical metabolism by CYP2E1, an isoform of cytochrome P450, forming 4HNE adducts [77,78]. Increased CYP2E1 activity led to significantly higher 4-HNE levels across cell types in the liver [79]. In biological systems where the 4HNE formation rate seems to be much slower than the EDC excretion rates (e.g., short-lived EDC with half-lives of elimination in the order of a few hours), 4HNE may not be used as a chrono-impacted marker in such a context, as also suggested in our study.

This study had some strengths and limitations. The use of repeatedly collected urine samples during the day, instead of a single spot urine void in the day, in analyzing exposure patterns and concentrations of the sample population to environmental compounds was a strength, as it allowed for observing the chrono-varying diurnal EDC biomarker variation and reduced the probability of exposure misclassification. Another strength was the simultaneous analysis of a suite of different EDC classes and various chemicals within each EDC class. However, there were also a few limitations. The lack of controlling for the time stamp of each food or PCP usage activity combined with varying half-lives of elimination for the different EDCs monitored made it difficult to match food/PCP exposures with the urinary EDC metabolites. An earlier analysis of the same study demonstrated that the dietary estimated exposure correlated better with the evening urinary phthalate metabolite levels [80]. The study population was not representative of EDC exposures of the Norwegian population based on demographics and dietary habits. Moreover, previous-day exposures, including dietary and PCP usage habits, were not recorded. The urination time stamps were matched with the group time stamps of eating and the use of PCP without having designed the study to consider the time needed for absorption and metabolism of the compounds before urinary excretion. 

More studies are needed to effectively control for the time of food intake in relation to the monitored diurnal variation in the biomarkers of exposure to non-persistent EDCs (with relatively short half-lives of elimination) and their downstream biological perturbations. It becomes obvious that the totality of environmental agents, such as chemicals and diet, including behaviors and lifestyles throughout one’s lifetime or in critical life stages, including the endogenous response, shall be characterized in detail if one were to further advance our understanding of the interactions occurring at the interface of the external and internal exposome domains [27]. This may be achieved with the methodological framework and tools of the human exposome concept that, by definition, incorporates the global dimensions of chronos and location [27,81,82]. 

## 5. Conclusions

Evidence obtained in this study suggests that the concentrations of some urinary EDCs metabolites followed a diurnal pattern (phthalates), while this was less consistent for the studied parabens and bisphenols. Diurnal (24-h) variation in urinary biomarkers of exposure may be particularly important for short-lived chemicals. The biological mechanism responsible for this interesting phenomenon is not yet clear; the influence of metabolism and associated time-dependent enzymatic activity patterns is possible, but more data are needed. The impact of exposure to EDCs, even at low concentrations, was shown to have a significant impact on lipid-based oxidative damage. The timing of human exposure to certain EDCs and the associated (chrono)toxicity window may be worth studying in more detail. The human exposome concept and its critical windows of susceptibility are key methodological concepts that would help in better designing such human chronotoxicity studies. To this extent, exposome studies with a longitudinal design are needed to further elucidate the human chronotoxicity potential of EDCs and the associated biological mechanisms and pathways.

## Figures and Tables

**Table 1:** Demographic characteristics of the study participants.

Basic Characteristics	Overall (*n* = 144)	Males (*n* = 44)	Females (*n* = 100)
Age (years, mean ± SD)	42.56± 12.05	43.36 ± 11.65	42.20 ± 12.26
Weight (kg, mean ± SD)	70.45 ± 11.71	81.98 ± 8.54	65.22 ± 8.88
Height (m, mean ± SD)	1.72 ± 0.08	1.81 ± 0.06	1.68 ± 0.06
BMI (kg/m^2^, mean ± SD)	23.47 ± 3.52	24.94 ± 2.33	22.82 ± 3.77
Smoking status (*n*, %)			
Non-smokers	90 (62.5)	26 (59.1)	64 (64)
Ex-smokers	35 (24.3)	11 (25.0)	24 (24)
Occasional smokers	19 (13.2)	7 (15.9)	12 (12)
Education (*n*, %)			
University/college			
up to 4 years	30 (20.8)	8 (18.2)	22 (22)
>4 years	114 (79.2)	36 (81.8)	78 (78)

**Table 2:** Percentiles of the specific gravity-adjusted biomarkers of exposure to EDCs and the biomarker of effect/lipid peroxidation (4HNE) by the time of urine sampling.

Biomarkers	Time Group	Min	5th	25th	50th	75th	90th	95th	Max
MeP (ug)	6:00–12:00	0.427	1.075	3.013	5.937	12.582	23.521	44.215	418
	12:00–18:00	0.406	0.815	1.751	3.872	8.524	18.277	34.046	1990
	18:00–6:00	0.607	1.923	4.099	8.219	18.517	34.438	60.88	364
	Overall	0.406		2.801	5.955	12.715			1990
MiBP (ug)	6:00–12:00	0.504	1.084	2.394	4.341	6.969	11.789	17.681	106
	12:00–18:00	0.11	0.822	1.54	2.569	5.005	7.906	10.947	23.5
	18:00–6:00	0.917	2.214	3.907	5.738	9.371	17.15	24.399	78.4
	Overall	0.110		2.289	4.370	7.340			106.4
MnBP (ug)	6:00–12:00	0.809	1.528	3.119	5.174	9.147	17.033	21.591	42.1
	12:00–18:00	0.479	0.979	2.129	3.431	5.378	9.061	11.97	20.6
	18:00–6:00	1.476	2.537	5.036	8.036	14.621	21.362	24.603	30.5
	Overall	0.480		2.995	5.192	9.531			42.1
MBzP (ug)	6:00–12:00	0.064	0.221	0.418	0.686	1.167	2.017	3.247	40.9
	12:00–18:00	0.043	0.133	0.308	0.478	0.818	1.505	2.174	8.7
	18:00–6:00	0.166	0.363	0.710	1.068	1.911	2.545	3.532	40.6
	Overall	0.043		0.425	0.733	1.248			40.9
SumDEHP (ug)	6:00–12:00	2.379	3.52	8.463	12.526	22.372	33.672	42.43	64.7
	12:00–18:00	1.455	3.079	6.041	9.537	14.741	26.007	35.354	176.8
	18:00–6:00	2.665	6.971	12.979	19.201	30.559	44.156	58.501	127.7
	Overall	1.455		8.467	13.295	23.315			176.8
SumDiNP (ug)	6:00–12:00	0.568	1.368	2.916	5.448	10.466	20.743	38.159	526
	12:00–18:00	0.661	1.187	2.435	4.805	7.998	14.915	29.127	156
	18:00–6:00	0.644	2.645	5.196	8.271	15.653	37.221	69.239	1276
	Overall	0.568		3.185	6.219	11.136			1276
SumDINCH (ug)	6:00–12:00	0.066	0.158	0.378	0.691	1.239	2.704	5.526	12.5
	12:00–18:00	0.025	0.12	0.295	0.483	0.957	1.637	2.345	7.8
	18:00–6:00	0.124	0.222	0.555	0.941	1.654	2.762	4.389	93.3
	Overall	0.025		0.399	0.710	1.216			93.3
oh-MPHP (ug)	6:00–12:00	0.005	0.02	0.078	0.196	0.381	0.582	0.771	2.7
	12:00–18:00	0.007	0.017	0.07	0.16	0.299	0.507	0.684	1.18
	18:00–6:00	0.018	0.038	0.135	0.309	0.538	0.851	1.315	5.95
	Overall	0.005		0.084	0.208	0.410			5.95
MEPA (ug/L)	6:00–12:00	0.816	1.512	3.394	6.305	37.192	99.038	158.275	18519
	12:00–18:00	0.336	1.11	2.954	5.85	24.273	81.163	134.853	3960
	18:00–6:00	0.756	1.456	3.277	7.320	27.653	87.744	123.885	6728
	Overall	0.336		3.246	6.467	25.362			18519
ETPA (ug/L)	6:00–12:00	0.024	0.164	0.520	1.235	3.432	14.118	26.195	761.7
	12:00–18:00	0.026	0.144	0.390	1.083	3.194	12.177	36.465	271.1
	18:00–6:00	0.029	0.145	0.348	0.841	3.269	19.471	40.127	2038
	Overall	0.024		0.422	1.083	3.264			2038
PRPA (ug/L)	6:00–12:00	0.008	0.012	0.024	0.251	2.827	20.076	46.206	384.9
	12:00–18:00	0.008	0.012	0.024	0.095	1.159	10.660	19.738	225.7
	18:00–6:00	0.008	0.012	0.027	0.169	3.404	16.416	32.363	175.7
	Overall	0.008		0.024	0.173	2.366			384.9
BUPA (ug/L)	6:00–12:00	0.014	0.018	0.032	0.065	0.179	0.528	1.083	9.82
	12:00–18:00	0.016	0.019	0.041	0.074	0.177	0.347	0.585	5.45
	18:00–6:00	0.014	0.019	0.032	0.051	0.162	0.364	0.681	47.1
	Overall	0.014		0.035	0.065	0.176			47.1
BPA (ug/L)	6:00–12:00	0.014	0.219	0.621	1.393	2.439	3.965	5.646	21.1
	12:00–18:00	0.011	0.060	0.602	1.059	2.175	4.176	4.983	24.6
	18:00–6:00	0.014	0.184	0.678	1.170	1.965	3.774	5.319	19.6
	Overall	0.011		0.621	1.163	2.194			24.6
BPS (ug/L)	6:00–12:00	<LOD	LOD	<LOD	<LOD	<LOD	0.501	1.083	2.8
	12:00–18:00	<LOD	<LOD	<LOD	<LOD	0.182	0.455	0.693	7.04
	18:00–6:00	<LOD	<LOD	<LOD	<LOD	0.165	0.691	1.082	55.7
	Overall	<LOD	<LOD	<LOD	<LOD	0.138			55.7
BPF (ug/L)	6:00–12:00	<LOD	<LOD	<LOD	<LOD	<LOD	<LOD	<LOD	6.0
	12:00–18:00	<LOD	<LOD	<LOD	<LOD	<LOD	<LOD	<LOD	7.7
	18:00–6:00	<LOD	<LOD	<LOD	<LOD	<LOD	<LOD	0.580	12.8
	Overall	<LOD		<LOD	<LOD	<LOD			12.8
4HNE (ug/L)	6:00–12:00	3.028	3.262	3.763	4.042	4.36	4.583	4.784	5.6
	12:00–18:00	2.766	3.289	3.813	4.083	4.458	4.771	5.026	5.8
	18:00–6:00	2.905	3.336	3.762	4.044	4.294	4.618	4.948	6.5
	Overall	2.766		3.775	4.048	4.375			6.5

**Table 3:** Mixed-effect models for the association between the SG-adjusted and logarithm transformed biomarkers of exposure to EDCs and the biomarker of effect (4HNE).

Log(4HNE)				
Parent EDCs	Biomarker	Estimate	95% CI	*q* Value
Phthalates	MeP	0.09	0.05–0.13	<0.001
	MiBP	0.14	0.09–0.20	<0.001
	MnBP	0.143	0.088–0.199	<0.001
	MBzP	0.111	0.059–0.163	<0.001
	SumDEHP	0.183	0.125–0.240	<0.001
	SumDiNP	0.103	0.061–0.146	<0.001
	SumDINCH	0.113	0.065–0.161	<0.001
	oh-MPHP	0.125	0.083–0.167	<0.001
Parabens	MEPA	0.13	0.10–0.17	<0.001
	ETPA	0.096	0.064–0.128	<0.001
	PRPA	0.039	0.017–0.061	<0.001
	BUPA	0.123	0.064–0.181	<0.001
Bisphenols	BPA	0.128	0.091–0.165	<0.001
	BPS	−0.015	−0.114–0.084	0.767

## Data Availability

All data are available upon request to the corresponding author via email (konstantinos.makris@cut.ac.cy).

## Supplementary Materials

The following supporting information can be downloaded at: https://www.mdpi.com/article/10.3390/toxics10040181/s1, Figure S1: Correlation matrix plot of the SG-adjusted, logarithm-transformed biomarkers of exposure to EDCs and the biomarker of effect or lipid peroxidation, 4HNE. From light blue to deep blue means positive correlation; from white to dark red means no or negative correlation; Figure S2: Box-plots for specific gravity-adjusted, logarithm-transformed values of EDCs and 4HNE per time group measured in urine samples; Figure S3: Visualization of the association between SG-adjusted, log-transformed EDCs and 4HNE with time group based on sex; Table S1: Phthalate metabolites determined in 24-hour urine of study day; Table S2: Environmental phenol and paraben substances determined in 24-hour urine of study day; Table S3: Median concentrations of the specific gravity-adjusted biomarkers of exposure to EDC and 4HNE by the time of urine sampling; Table S4 (i)–(xv): Linear mixed-effect models of specific gravity-adjusted biomarker of exposure & effect as a function of time of urine sampling in the day adjusted for dietary habits and personal care product use (reference: noon); Table S5 (i)–(xiv): Linear mixed-effect models for the association between the specific gravity-adjusted log(4HNE) and logarithm-transformed biomarker of exposure.

## Abbreviations

4HNE, 4-Hydroxynonenal; BBzP, butyl benzyl phthalate; BMI, body mass index; BPA, bisphenol A; BPF, bisphenol F; BPs, bisphenols; BPS, bisphenol S; BUPA, butyl paraben; CES, carboxylesterases; CYPs, cytochromes P450; cx-MiNP, mono-4-methyl-7- carboxyoctyl phthalate; DEHP, diethyl phthalate; DEP, diethyl phthalate; DiBP, di-iso-butyl phthalate; DINCH, 1,2-cyclohexane dicarboxylic acid diisononyl ester; DiNP, di-iso-nonyl phthalate; DnBP, di-n-butyl phthalate; DPHP, di(2-propyl heptyl) phthalate; EDC, endocrine-disrupting chemical; ETPA, ethyl paraben; FDR, false discovery rate; FFQ, food frequency questionnaire; HNE-MA, 4-hydroxy-2-nonenal-mercapturic acid; ICC, intraclass correlation coefficient; LME, linear mixed-effect; LOD, limit of detection; LOQ, limit of quantification; MBzP, mono benzyl phthalate; MECPP, Mono-2-ethyl 5-carboxypentyl phthalate; MEHHP, mono-2-ethyl-5-hydroxyhexyl phthalate; MEHP, mono-2-ethylhexyl phthalate; MEOHP, mono-2-ethyl-5-oxohexyl phthalate; MEP, monoethyl phthalate; MiBP, mono-isobutyl phthalate; MMCHP, mono-2-carboxymethyl hexyl phthalate; MnBP, mono-n-butyl phthalate; MEP, methylparaben; OHdG, 8-hydroxy-2′-deoxyguanosine, 8-; OH-EtP, ethyl-protocatechuic acid; OH-MeP, methyl-protocatechuic acid; oh-MINCH, (2-(((Hydroxy-4-methyloctyl)oxy)carbonyl)-cyclohexane carboxylic acid; oh-MiNP, mono-4-methyl-7-hydroxyoctyl phthalate; oh-MPHP, 6-hydroxy monopropylheptyl phthalate; oxo-MINCH, 2-(((4-Methyl-7-oxyooctyl)oxy)carbonyl)-cyclohexanecarboxylic acid; oxo-MiNP, mono-4-methyl-7-oxooctyl phthalate; PBs, parabens; PCP, personal care product; PVC, Polyvinyl chloride; SCN, suprachiasmatic nucleus; SD, standard deviation; SG, specific gravity; THM, trihalomethanes.
