# Supplementary material for: Diurnal Variation in Biomarkers of Exposure to Endocrine-Disrupting Chemicals and Their Association with Oxidative Damage in Norwegian Adults: The EuroMix Study
Source: Toxics. 2022 Apr 7;10(4):181. doi: 10.3390/toxics10040181 (PMC9028082; doi:10.3390/toxics10040181)
Supplement: Supplementary file 1 [file toxics-10-00181-s001.zip › toxics-1647696-supplementary.pdf]

toxics

MDPI

Supplementary Materials: Diurnal Variation in Biomarkers of Exposure to Endocrine-Disrupting Chemicals and Their Association with Oxidative Damage in Norwegian Adults: The EuroMix Study

Samuel Olushola Abimbola, Christina Xeni, Amrit Kaur Sakhi, Behzad Heibati, Trine Husøy, Hubert Dirven and Konstantinos C. Makris

Table S1. Phthalate metabolites determined in 24-hour urine of study day.

**Phthalate**		**Phthalate metabolites**		**LOD** µg/ml	**LOQ** µg/ml	**Samples < LOD (%)**
Diethyl phthalate	DEP	Monoethyl phthalate	MEP	0.20	0.50	0
Di-iso-butyl phthalate	DiBP	Mono-iso-butyl phthalate	MiBP	0.20	0.50	0
Di-n-butyl phthalate	DnBP	Mono-n-butyl phthalate	MnBP	0.20	0.50	0
Butyl benzyl phthalate	BBzP	Mono benzyl phthalate	MBzP	0.07	0.20	0
Di(2-ethylhexyl) phthalate	DEHP	Mono-2-ethylhexyl phthalate	MEHP	0.20	0.50	12
		Mono-2-ethyl-5-hydroxyhexyl phthalate	MEHHP	0.20	0.50	0
		Mono-2-ethyl-5-oxohexyl phthalate	MEOHP	0.20	0.50	0
		Mono-2-ethyl 5-carboxypentyl phthalate	MECPP	0.70	2.00	0
		Mono-2-carboxymethyl hexyl phthalate	MMCHP	0.70	2.00	3
Di-iso-nonyl phthalate	DiNP	Mono-4-methyl-7-hydroxyoctyl phthalate	oh-MiNP	0.10	0.25	0
		Mono-4-methyl-7-oxooctyl phthalate	oxo-MiNP	0.10	0.25	0
		Mono-4-methyl-7-carboxyoctyl phthalate	cx-MiNP	0.40	1.00	0
1,2-cyclohexane dicarboxylic acid diisononyl ester (phthalate substitute)	DINCH	2-(((Hydroxy-4-methyloctyl)oxy)carbonyl)-cyclohexanecarboxylic Acid	oh-MINCH	0.07	0.20	11
		2-(((4-Methyl-7-oxyooctyl)oxy)carbonyl)-cyclohexanecarboxylic Acid	oxo-MINCH	0.07	0.20	4
Di(2-propyl heptyl) *phthalate*	DPHP	6-Hydroxy Monopropylheptylphthalate	oh-MPHP	0.07	0.20	24

LOD – limit of detection, LOQ – limit of quantification.

Table S2. Environmental phenol and paraben substances determined in 24-hour urine of study day.

**Compounds**	**Abbreviations**	**LOD µg/ml**	**LOQ µg/ml**	**Samples < LOD (%)**
Methyl paraben	MEPA	0.04	0.10	0
Ethyl paraben	ETPA	0.04	0.10	1
Propyl paraben	PRPA	0.04	0.10	35
Butyl paraben	BUPA	0.07	0.20	50
Bisphenol A	BPA	0.04	0.10	4
Bisphenol S	BPS	0.10	0.40	71
Bisphenol F	BPF	0.07	0.20	96

LOD – limit of detection, LOQ – limit of quantification.

Table S1: Median concentrations of the specific gravity-adjusted biomarkers of exposure to EDC and 4HNE by the time of urine sampling.

Biomarkers	Time Group	Median
MEP	6:00-12:00	5.937
	12:00-18:00	3.872
	18:00-6:00	8.219
MiBP	6:00-12:00	4.341
	12:00-18:00	2.569
	18:00-6:00	5.738
MnBP	6:00-12:00	5.174
	12:00-18:00	3.431
	18:00-6:00	8.036
MBzP	6:00-12:00	0.686
	12:00-18:00	0.478
	18:00-6:00	1.068
SumDEHP	6:00-12:00	12.526
	12:00-18:00	9.537
	18:00-6:00	19.201
SumDiNP	6:00-12:00	5.448
	12:00-18:00	4.805
	18:00-6:00	8.271
SumDINCH	6:00-12:00	0.691
	12:00-18:00	0.483
	18:00-6:00	0.941
oh.MPHP	6:00-12:00	0.196
	12:00-18:00	0.160
	18:00-6:00	0.309
MEPA	6:00-12:00	6.305
	12:00-18:00	5.850
	18:00-6:00	7.320
ETPA	6:00-12:00	1.235
	12:00-18:00	1.083
	18:00-6:00	0.841
PRPA	6:00-12:00	0.251
	12:00-18:00	0.095
	18:00-6:00	0.169
BUPA	6:00-12:00	0.065
	12:00-18:00	0.074
	18:00-6:00	0.051
BPA	6:00-12:00	1.393
	12:00-18:00	1.059
	18:00-6:00	1.170
BPS	6:00-12:00	<LOD
	12:00-18:00	<LOD
	18:00-6:00	<LOD
BPF	6:00-12:00	<LOD
	12:00-18:00	<LOD
	18:00-6:00	<LOD
4HNE	6:00-12:00	4.042
	12:00-18:00	4.083
	18:00-6:00	4.044

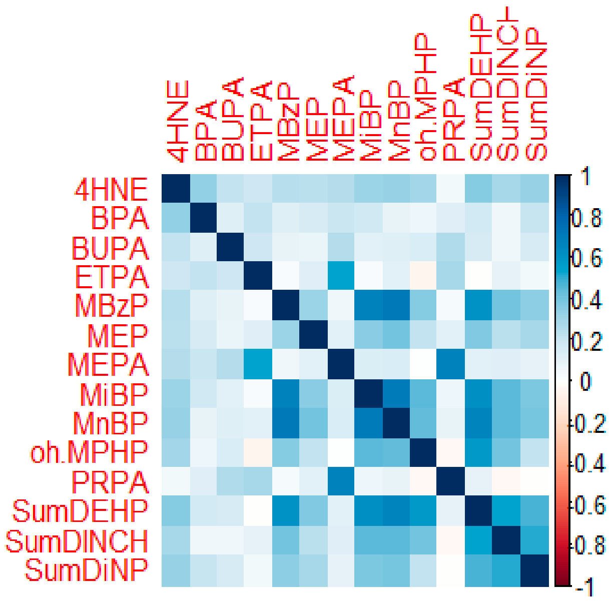

Figure S1. Correlation matrix plot of the SG-adjusted, logarithm-transformed biomarkers of exposure to EDCs and the biomarker of effect or lipid peroxidation, 4HNE. From light blue to deep blue means positive correlation; from white to dark red means no or negative correlation.

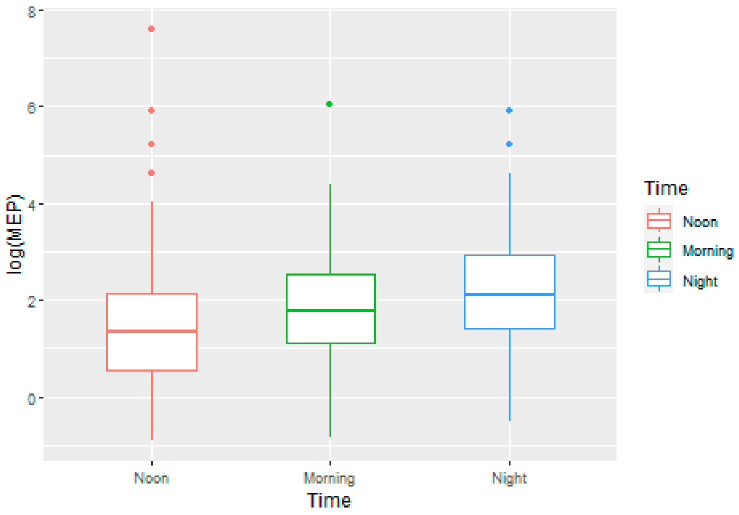

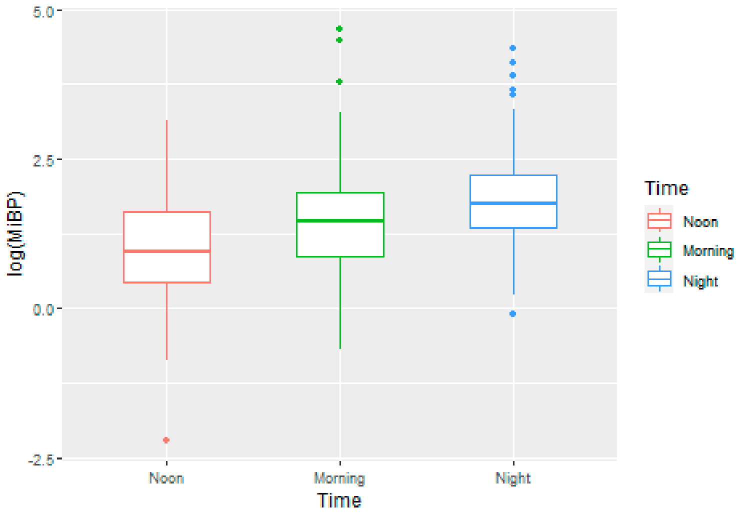
5.0
2.5
0.0
-2.5
log(MiBP)
Noon
Morning
Night
Time
Time
Noon
Morning
Night

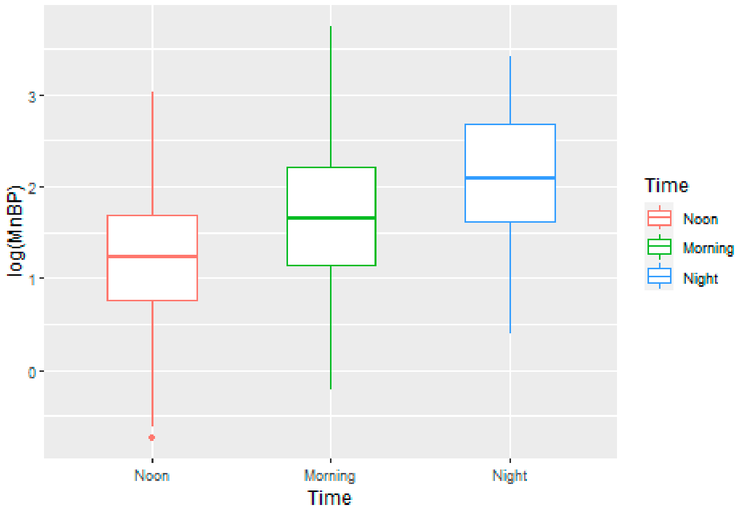
3
2
1
0
log(MnBP)
Noon
Morning
Night
Time
Time
Noon
Morning
Night

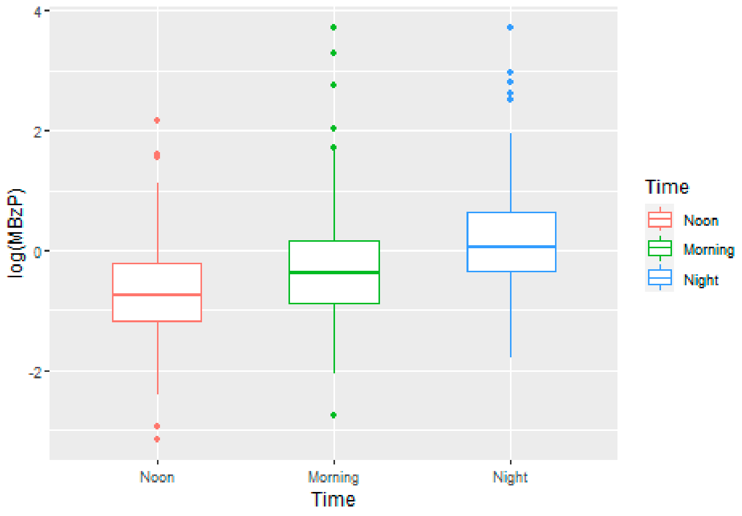
4
2
0
-2
log(MBzP)
Noon
Morning
Night
Time
Time
Noon
Morning
Night

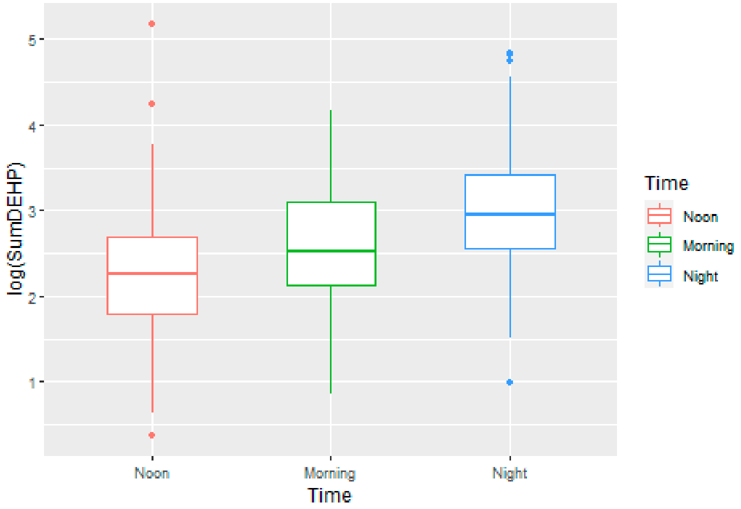
log(SumDEHP)
5
4
3
2
1
Noon
Morning
Night
Time
Time
Noon
Morning
Night

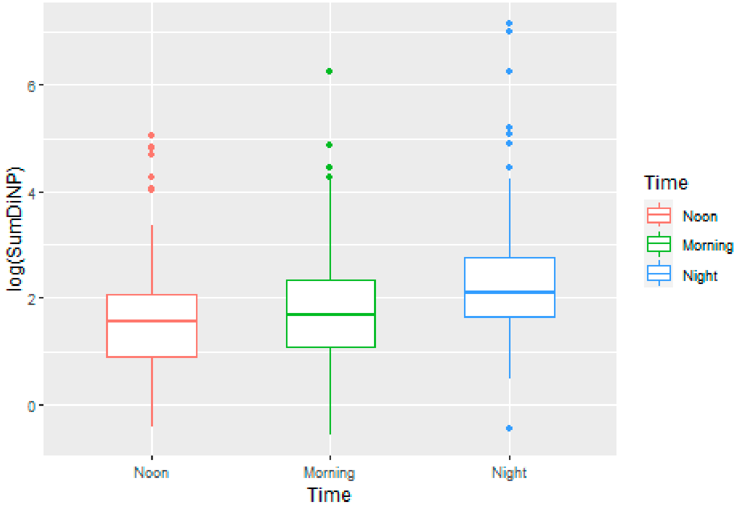
log(SumDiNP)
6
4
2
0
Noon
Morning
Night
Time
Time
Noon
Morning
Night

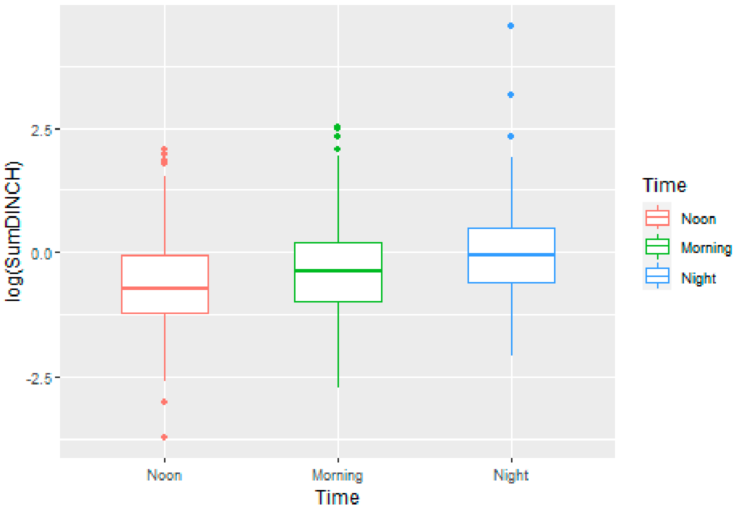
log(SumDINCH)
2.5
0.0
-2.5
Noon
Morning
Night
Time
Time
Noon
Morning
Night

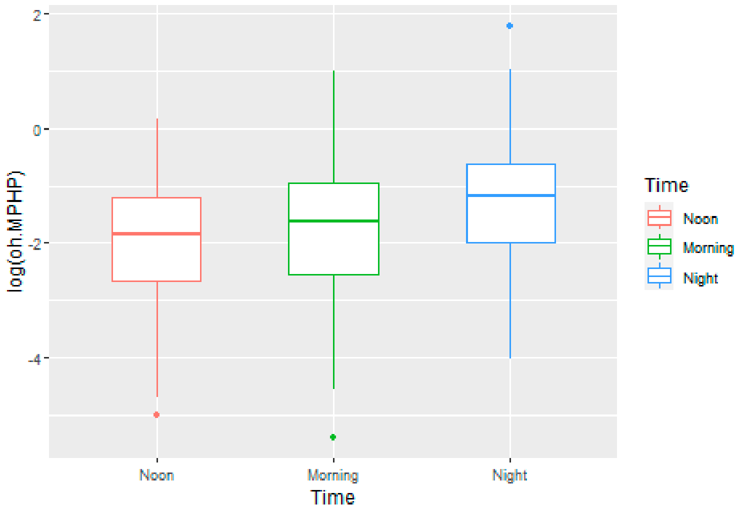
log(oh.MPHP)
2
0
-2
-4
Noon
Morning
Night
Time
Time
Noon
Morning
Night

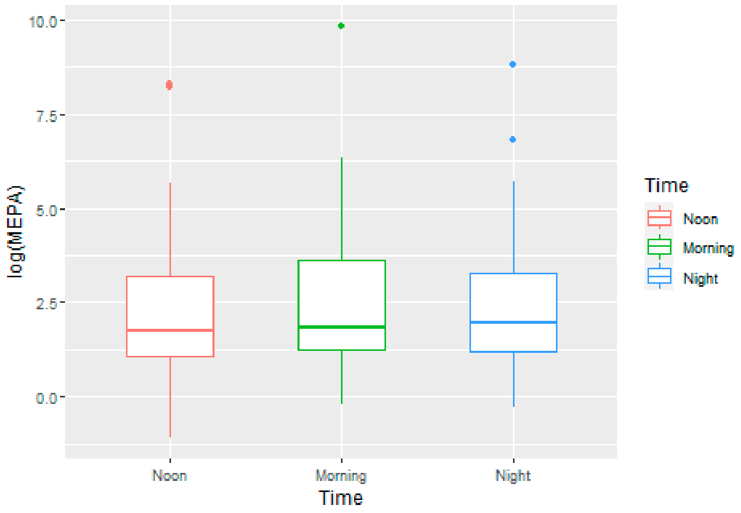
log(MEPA)
10.0
7.5
5.0
2.5
0.0
Noon
Morning
Night
Time
Time
Noon
Morning
Night

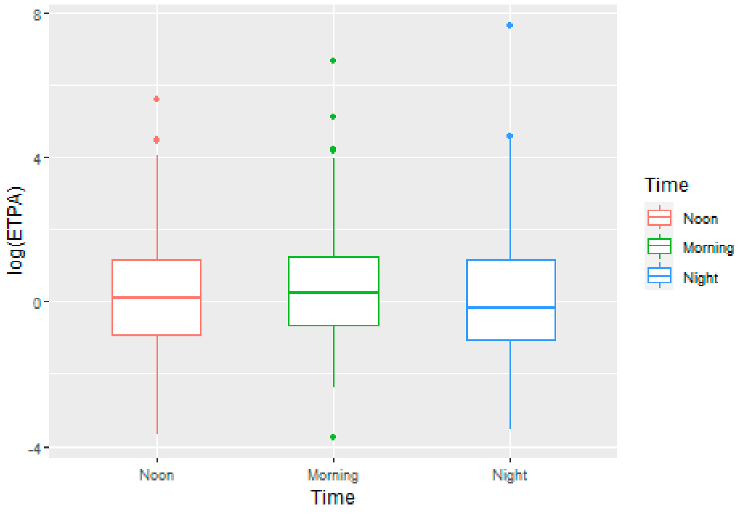
log(ETPA)
8
4
0
-4
Noon
Morning
Night
Time
Time
Noon
Morning
Night

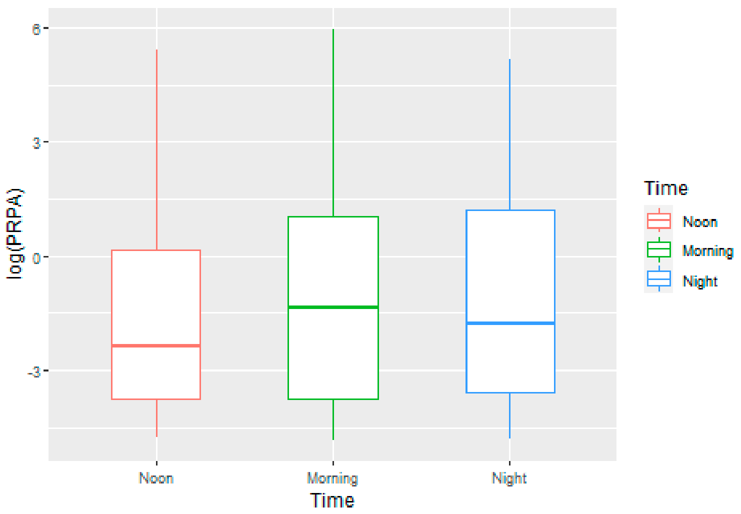
6
3
0
-3
log(PRPA)
Noon
Morning
Night
Time
Time
Noon
Morning
Night

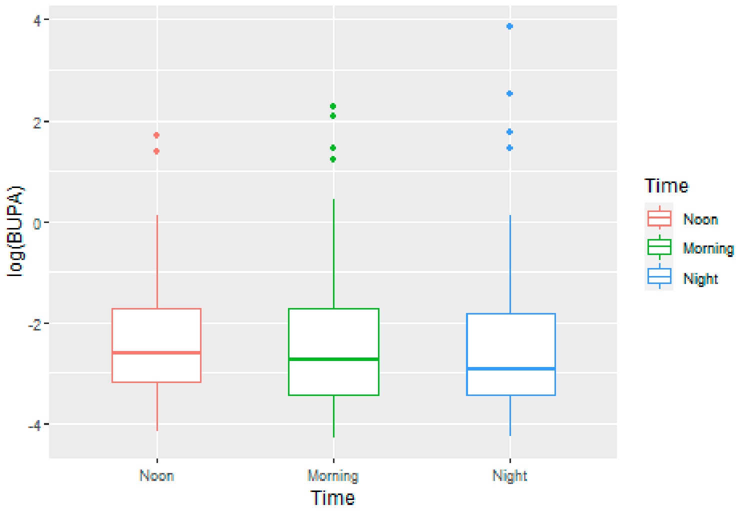
4
2
0
-2
-4
log(BUPA)
Noon
Morning
Night
Time
Time
Noon
Morning
Night

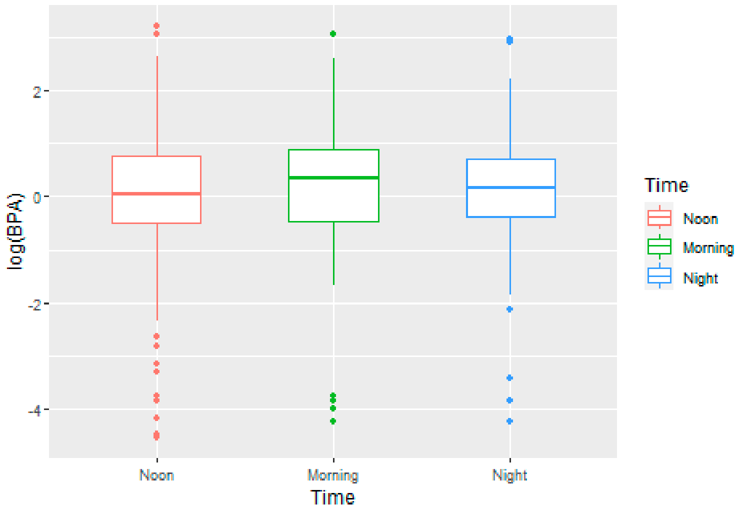
2
0
-2
-4
log(BPA)
Noon
Morning
Night
Time
Time
Noon
Morning
Night

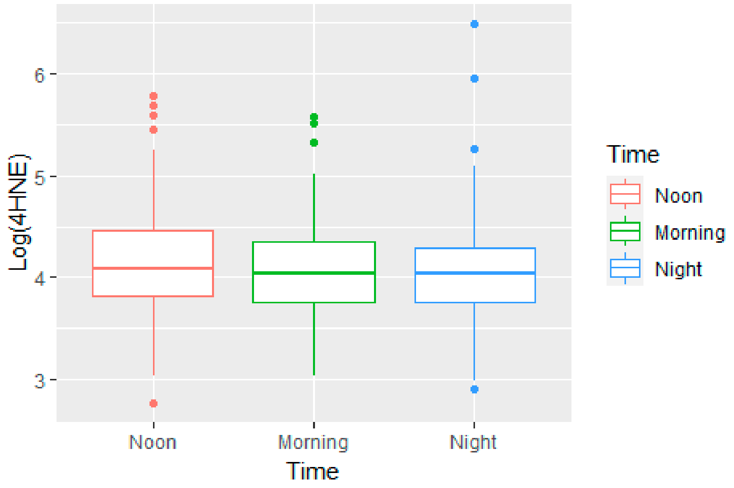

Figure S2. Box-plots for specific gravity-adjusted, logarithm-transformed values of EDCs and 4HNE per time group measured in urine samples.

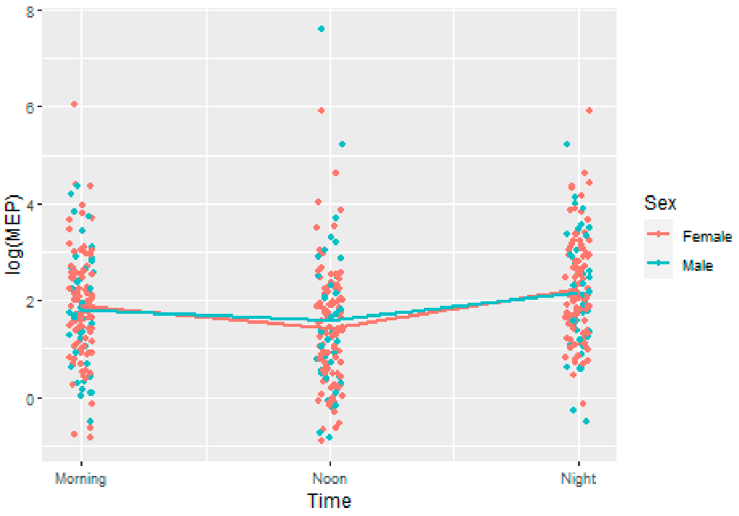

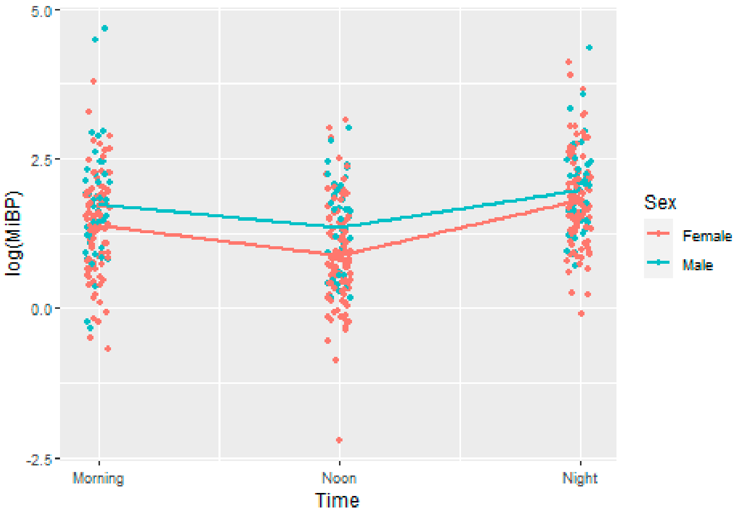

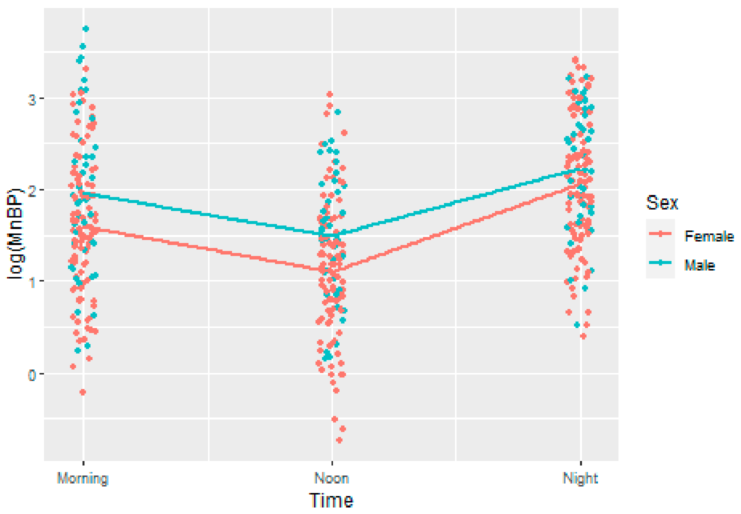
log(MnBP)
3
2
1
0
Morning
Noon
Night
Time
Sex
Female
Male

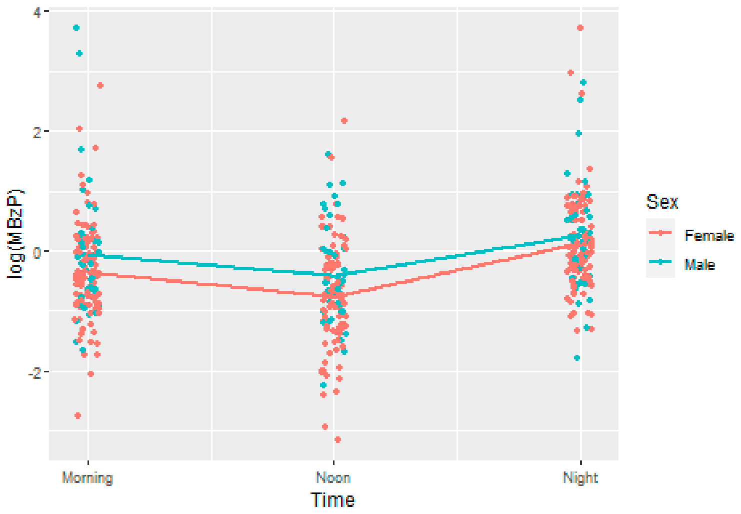
log(MBzP)
4
2
0
-2
Morning
Noon
Night
Time
Sex
Female
Male

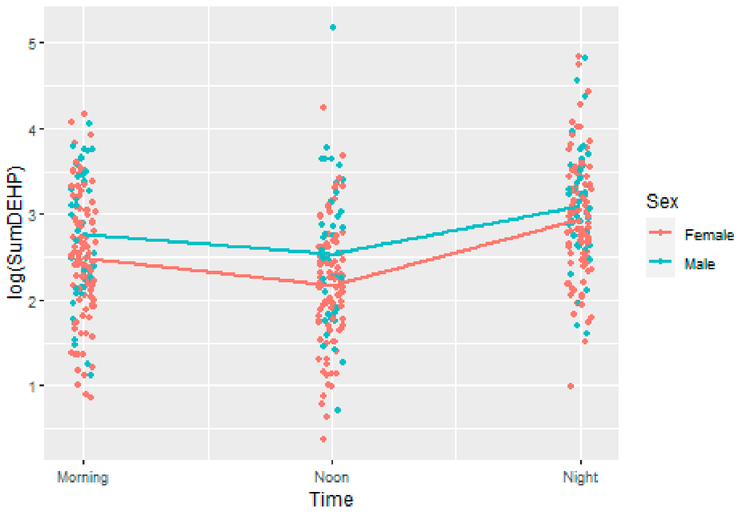
log(SumDEHP)
5
4
3
2
1
Morning
Noon
Night
Time
Sex
Female
Male

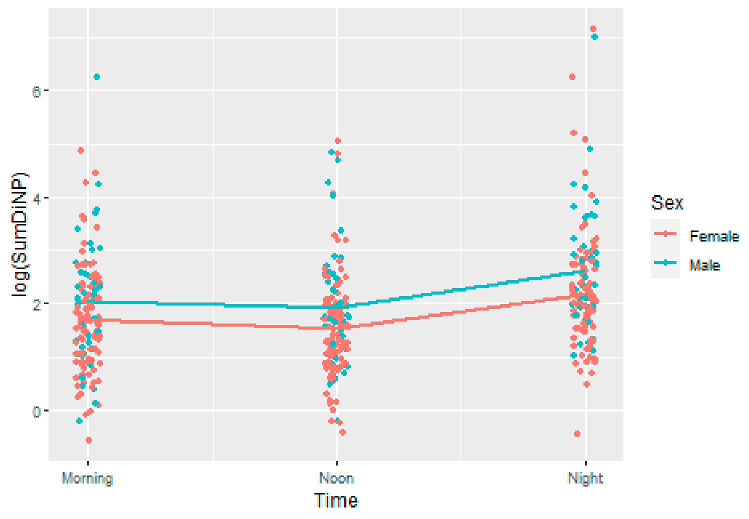
log(SumDiNP)
6
4
2
0
Morning
Noon
Night
Time
Sex
Female
Male

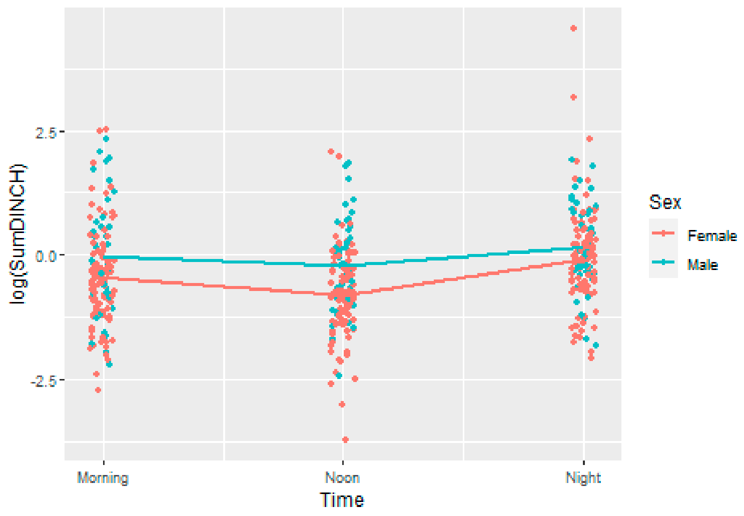
log(SumDINCH)
2.5
0.0
-2.5
Morning
Noon
Night
Time
Sex
Female
Male

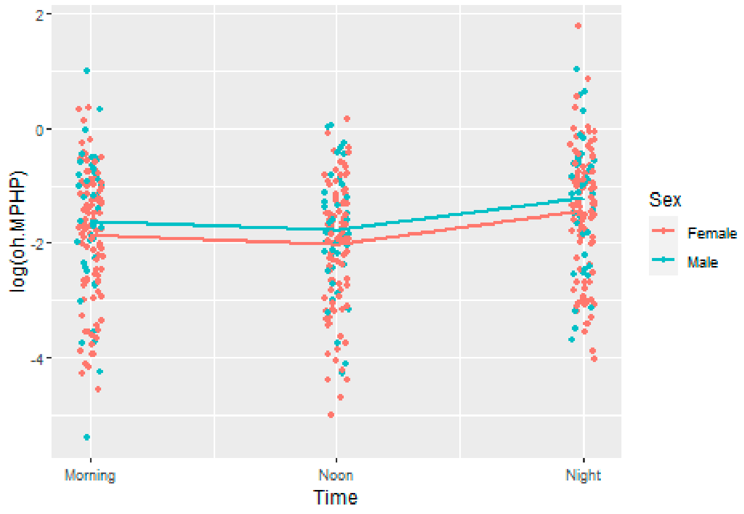
log(oh.MPHP)
2
0
-2
-4
Morning
Noon
Night
Time
Sex
Female
Male

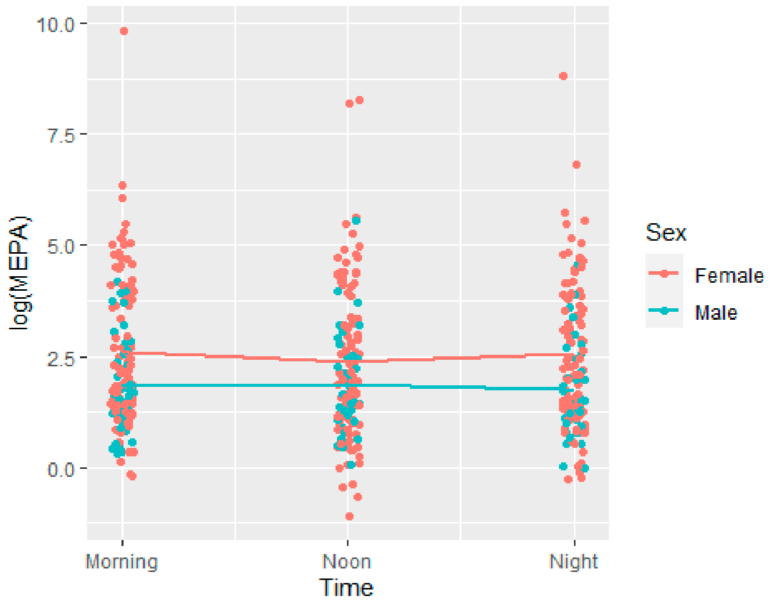
log(MEPA)
10.0
7.5
5.0
2.5
0.0
Morning
Noon
Night
Time
Sex
Female
Male

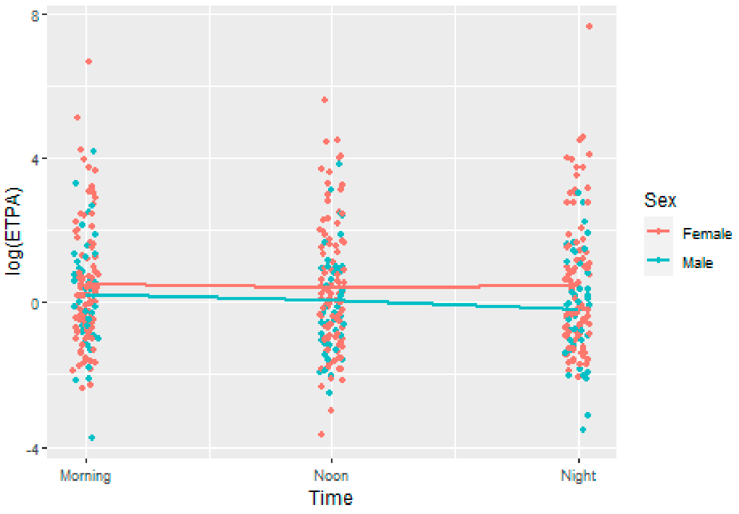
log(ETPA)
8
4
0
-4
Morning
Noon
Night
Time
Sex
Female
Male

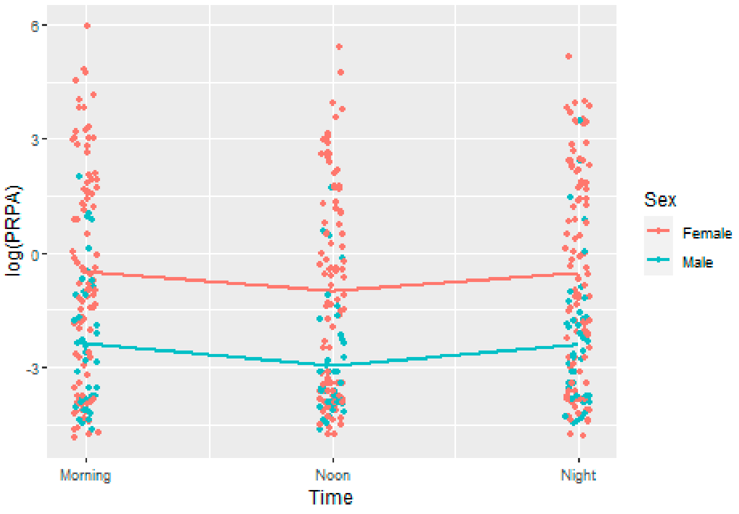
log(PRPA)
6
3
0
-3
Morning
Noon
Night
Time
Sex
Female
Male

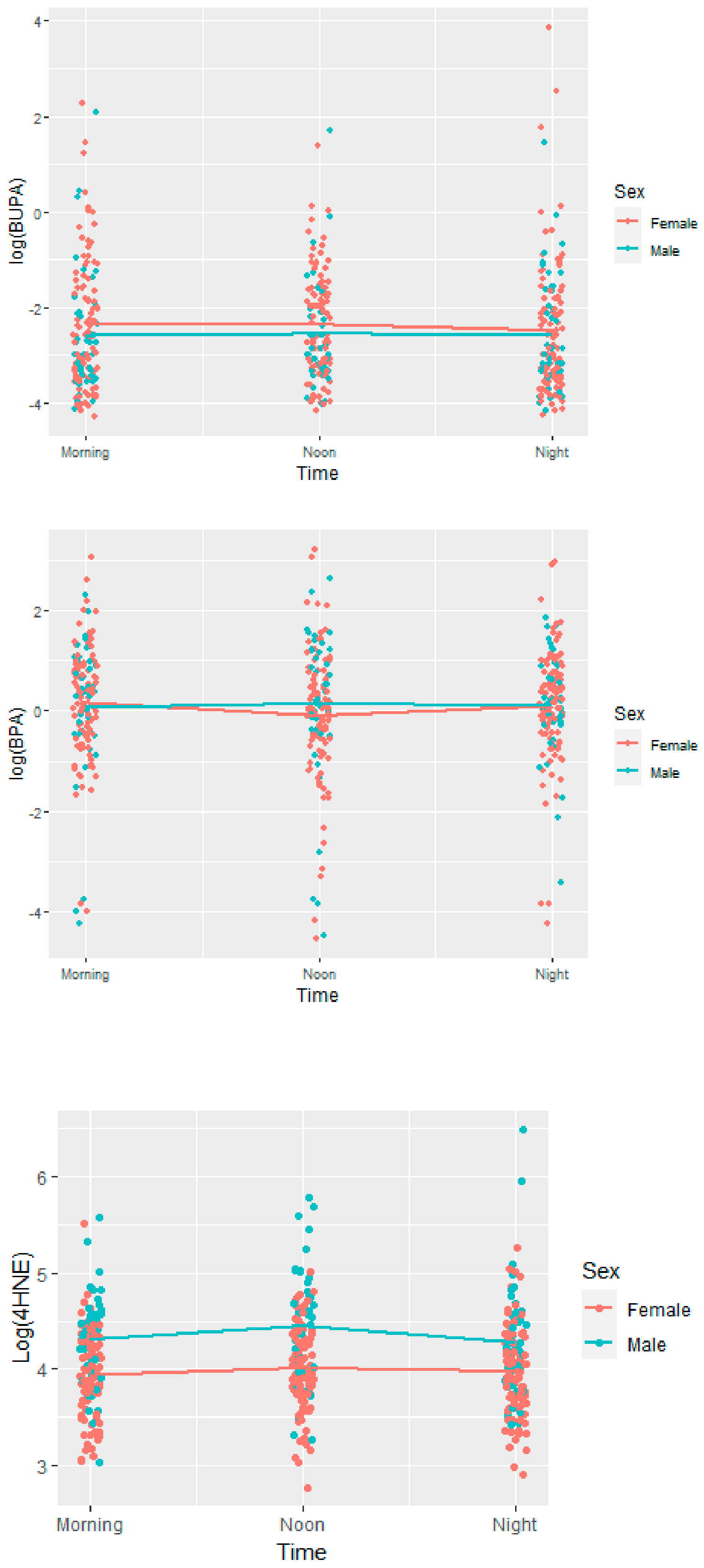

Figure S3. Visualization of the association between SG-adjusted, log-transformed EDCs and 4HNE with time group based on sex.

Table S4. i): Linear mixed-effect models of specific gravity-adjusted MiBP as a function of time of urine sampling in the day adjusted for dietary habits and personal care product use (reference: noon).

	log(MiBP)			
Predictors	Estimates	CI	p	p adjusted
(Intercept)	1.19	0.24 – 2.14	0.014	
Time [Morning]	0.32	0.15 – 0.49	**<0.001**	**<0.001**
Time [Night]	0.71	0.55 – 0.87	**<0.001**	**<0.001**
fruit [Yes]	-0.00	-0.13 – 0.12	0.957	
others [Yes]	-0.01	-0.14 – 0.11	0.812	
sweets [Yes]	-0.00	-0.13 – 0.12	0.973	
butterandOil [Yes]	0.05	-0.08 – 0.18	0.430	
shower gel [Yes]	-0.04	-0.17 – 0.10	0.579	
hand cream [Yes]	-0.23	-0.53 – 0.08	0.144	
toothpaste [Yes]	0.17	0.01 – 0.32	**0.035**	0.154
anti wrinkle cream [Yes]	0.18	-0.22 – 0.57	0.382	
shaving products [Yes]	0.15	-0.43 – 0.74	0.607	
Age	0.01	-0.00 – 0.02	0.292	
Sex [Male]	0.38	0.11 – 0.65	**0.006**	**0.030**
BMI	-0.02	-0.06 – 0.01	0.225	

N.B.: Bold fonts indicate statistical significance (p/q value < 0.05).

Table S4. (ii): Linear mixed-effect models of specific gravity-adjusted MnBP as a function of time of urine sampling in the day adjusted for dietary habits and personal care product use (reference: noon).

	log(MnBP)			
Predictors	Estimates	CI	p	q value
(Intercept)	0.94	0.12 – 1.77	0.025	
Time [Morning]	0.38	0.21 – 0.55	**<0.001**	**<0.001**
Time [Night]	0.80	0.65 – 0.96	**<0.001**	**<0.001**
fruit [Yes]	0.03	-0.09 – 0.15	0.590	
others [Yes]	0.00	-0.12 – 0.12	0.978	
sweets [Yes]	0.04	-0.08 – 0.16	0.560	
butterandOil [Yes]	0.02	-0.11 – 0.15	0.762	
shower gel [Yes]	-0.02	-0.15 – 0.12	0.806	
hand cream [Yes]	-0.17	-0.47 – 0.13	0.258	
toothpaste [Yes]	0.12	-0.03 – 0.27	0.130	
anti wrinkle cream [Yes]	0.05	-0.33 – 0.44	0.788	
shaving products [Yes]	0.16	-0.41 – 0.73	0.586	
Age	0.01	0.00 – 0.02	**0.005**	**0.026**
Sex [Male]	0.33	0.10 – 0.56	**0.006**	**0.030**
BMI	-0.02	-0.05 – 0.01	0.303	

N.B.: Bold fonts indicate statistical significance (p/q value < 0.05).

Table S4. (iii): Linear mixed-effect models of specific gravity-adjusted MBzP as a function of time of urine sampling in the day adjusted for dietary habits and personal care product use (reference: noon).

	log(MBzP)			
Predictors	Estimates	CI	p	q value
(Intercept)	-0.15	-1.14 – 0.83	0.761	
Time [Morning]	0.32	0.14 – 0.50	**0.001**	**0.006**
Time [Night]	0.76	0.59 – 0.93	**<0.001**	**<0.001**
fruit [Yes]	-0.03	-0.16 – 0.10	0.667	
others [Yes]	0.05	-0.08 – 0.18	0.413	
sweets [Yes]	0.06	-0.07 – 0.19	0.395	
butterandOil [Yes]	-0.00	-0.14 – 0.14	0.985	
shower gel [Yes]	-0.02	-0.17 – 0.12	0.754	
hand cream [Yes]	-0.19	-0.52 – 0.13	0.242	
toothpaste [Yes]	0.10	-0.07 – 0.26	0.257	
anti wrinkle cream [Yes]	0.05	-0.37 – 0.47	0.800	
shaving products [Yes]	0.21	-0.41 – 0.84	0.498	
Age	-0.01	-0.02 – 0.01	0.337	
Sex [Male]	0.29	0.01 – 0.56	**0.044**	0.194
BMI	-0.02	-0.05 – 0.02	0.355	

N.B.: Bold fonts indicate statistical significance (p/q value < 0.05).

Table S4. (iv): Linear mixed-effect models of specific gravity-adjusted SumDEHP as a function of time of urine sampling in the day adjusted for dietary habits and personal care product use (reference: noon).

	log(SumDEHP)			
Predictors	Estimates	CI	p	q value
(Intercept)	2.37	1.62 – 3.11	**<0.001**	
Time [Morning]	0.30	0.11 – 0.48	**0.002**	**0.011**
Time [Night]	0.71	0.54 – 0.88	**<0.001**	**<0.001**
fruit [Yes]	0.02	-0.10 – 0.15	0.730	
others [Yes]	0.03	-0.10 – 0.16	0.626	
sweets [Yes]	0.02	-0.11 – 0.15	0.783	
butterandOil [Yes]	0.03	-0.11 – 0.16	0.666	
shower gel [Yes]	-0.13	-0.28 – 0.01	0.070	
hand cream [Yes]	-0.16	-0.47 – 0.16	0.325	
toothpaste [Yes]	0.02	-0.15 – 0.18	0.821	
anti wrinkle cream [Yes]	0.24	-0.18 – 0.65	0.262	
shaving products [Yes]	0.20	-0.42 – 0.82	0.525	
Age	-0.00	-0.01 – 0.01	0.942	
Sex [Male]	0.28	0.07 – 0.49	**0.008**	**0.043**
BMI	-0.01	-0.04 – 0.02	0.556	

N.B.: Bold fonts indicate statistical significance (p/q value < 0.05).

Table S4. (v): Linear mixed-effect models of specific gravity-adjusted SumDiNP as a function of time of urine sampling in the day adjusted for dietary habits and personal care product use (reference: noon) .

	log(SumDiNP)			
Predictors	Estimates	CI	p	q value
(Intercept)	1.46	0.29 – 2.63	**0.015**	
Time [Morning]	0.23	-0.02 – 0.48	0.076	
Time [Night]	0.73	0.50 – 0.97	**<0.001**	**<0.001**
Age	-0.01	-0.02 – 0.00	0.221	
Sex [Male]	0.38	0.05 – 0.71	**0.022**	0.103
BMI	0.01	-0.03 – 0.06	0.593	
fruit [Yes]	0.14	-0.04 – 0.32	0.116	
others [Yes]	0.08	-0.10 – 0.26	0.377	
sweets [Yes]	0.03	-0.15 – 0.22	0.710	
butterandOil [Yes]	-0.00	-0.19 – 0.19	0.983	
shower gel [Yes]	-0.07	-0.27 – 0.13	0.470	
hand cream [Yes]	-0.17	-0.61 – 0.27	0.454	
toothpaste [Yes]	-0.05	-0.28 – 0.18	0.654	
anti wrinkle cream [Yes]	0.15	-0.43 – 0.73	0.618	
shaving products [Yes]	-0.46	-1.32 – 0.40	0.293	

N.B.: Bold fonts indicate statistical significance (p/q value < 0.05).

Table S4. (vi): Linear Mixed-effect modelsof specific gravity-adjusted SumDINCH as a function of time of urine sampling in the day adjusted for dietary habits and personal care product use (reference: noon).

	**log(SumDINCH)**			
Predictors	Estimates	CI	p	q value
(Intercept)	-0.05	-1.13 – 1.04	0.931	
Time [Morning]	0.27	0.05 – 0.49	**0.017**	0.078
Time [Night]	0.58	0.37 – 0.79	**<0.001**	**<0.001**
fruit [Yes]	-0.04	-0.20 – 0.11	0.580	
others [Yes]	-0.00	-0.16 – 0.15	0.968	
sweets [Yes]	0.05	-0.11 – 0.20	0.569	
butterandOil [Yes]	0.00	-0.16 – 0.17	0.981	
shower.gel [Yes]	-0.04	-0.22 – 0.13	0.632	
hand.cream [Yes]	-0.20	-0.58 – 0.19	0.323	
toothpaste [Yes]	0.06	-0.14 – 0.26	0.540	
anti.wrinkle.cream [Yes]	0.05	-0.46 – 0.55	0.861	
shaving.products [Yes]	-0.11	-0.86 – 0.64	0.768	
Age	-0.00	-0.01 – 0.01	0.672	
Sex [Male]	0.49	0.18 – 0.79	**0.002**	**0.011**
BMI	-0.03	-0.07 – 0.01	0.192	

N.B.: Bold fonts indicate statistical significance (p/q value < 0.05).

Table S4. (vii): Linear mixed-effect models of specific gravity-adjusted oh.MPHP as a function of time of urine sampling in the day adjusted for dietary habits and personal care product use (reference: noon).

	log(oh.MPHP)			
Predictors	Estimates	CI	p	q value
(Intercept)	-1.78	-3.08 – -0.48	**0.007**	
Time [Morning]	0.19	-0.05 – 0.43	0.113	
Time [Night]	0.62	0.40 – 0.85	**<0.001**	**<0.001**
fruit [Yes]	0.03	-0.15 – 0.20	0.768	
others [Yes]	-0.01	-0.18 – 0.16	0.883	
sweets [Yes]	0.02	-0.16 – 0.19	0.857	
butterandOil [Yes]	0.02	-0.16 – 0.21	0.796	
shower.gel [Yes]	-0.06	-0.25 – 0.13	0.527	
hand.cream [Yes]	-0.00	-0.43 – 0.43	0.994	
toothpaste [Yes]	-0.09	-0.31 – 0.12	0.394	
anti.wrinkle.cream [Yes]	0.56	0.00 – 1.11	**0.049**	0.204
shaving.products [Yes]	0.09	-0.73 – 0.91	0.834	
Age	0.00	-0.01 – 0.02	0.716	
Sex [Male]	0.27	-0.09 – 0.64	0.144	
BMI	-0.02	-0.06 – 0.03	0.532	

N.B.: Bold fonts indicate statistical significance (p/q value < 0.05).

Table S4. **(viii):** Linear mixed-effect models of specific gravity-adjusted MEP as a function of time of urine sampling in the day adjusted for dietary habits and personal care product use (reference: noon).

	Log(MEP)			
Predictors	Estimates	CI	p	q value
(Intercept)	1.74	0.33 – 3.14	**0.015**	
Time [Morning]	0.39	0.16 – 0.61	**0.001**	**0.006**
Time [Night]	0.73	0.52 – 0.94	**<0.001**	**<0.001**
fruit [Yes]	0.02	-0.14 – 0.19	0.764	
others [Yes]	-0.06	-0.22 – 0.10	0.483	
sweets [Yes]	0.01	-0.15 – 0.17	0.915	
butterandOil [Yes]	0.05	-0.12 – 0.23	0.538	
shower gel [Yes]	-0.04	-0.22 – 0.14	0.639	
hand cream [Yes]	-0.12	-0.53 – 0.29	0.570	
toothpaste [Yes]	-0.00	-0.21 – 0.20	0.966	
anti wrinkle cream [Yes]	0.13	-0.39 – 0.66	0.616	
shaving products [Yes]	-0.34	-1.12 – 0.44	0.392	
Age	0.01	-0.01 – 0.02	0.290	
Sex [Male]	0.07	-0.32 – 0.47	0.718	
BMI	-0.03	-0.08 – 0.03	0.316	

N.B.: Bold fonts indicate statistical significance (p/q value < 0.05).

Table S4 (ix): Linear mixed-effect models of specific gravity-adjusted MEPA as a function of time of urine sampling in the day adjusted for dietary habits and personal care product use (reference: noon).

log(MEPA)				
Predictors	Estimates	CI	p	q value
(Intercept)	1.26	-0.61 – 3.13	0.186	
Time [Morning]	0.11	-0.06 – 0.29	0.190	
Time [Night]	0.08	-0.08 – 0.25	0.311	
meat [Yes]	-0.05	-0.23 – 0.13	0.559	
breads [Yes]	0.06	-0.13 – 0.24	0.554	
beverages [Yes]	-0.00	-0.32 – 0.31	0.977	
butterandOil [Yes]	0.04	-0.15 – 0.23	0.691	
Age	0.03	0.01 – 0.05	**0.007**	**0.040**
Sex [Male]	-0.71	-1.24 – -0.18	**0.008**	**0.043**
BMI	-0.00	-0.07 – 0.07	0.996	

N.B.: Bold fonts indicate statistical significance (p/q value < 0.05).

Table S4. (x): Linear mixed-effect models of specific gravity-adjusted ETPA as a function of time of urine sampling in the day adjusted for dietary habits and personal care product use (reference: noon).

log(ETPA)				
Predictors	Estimates	CI	p	q value
(Intercept)	-0.48	-2.51 – 1.55	0.642	
Time [Morning]	0.06	-0.16 – 0.28	0.603	
Time [Night]	-0.01	-0.22 – 0.20	0.917	
meat [Yes]	-0.01	-0.23 – 0.22	0.961	
breads [Yes]	0.15	-0.09 – 0.38	0.217	
beverages [Yes]	0.25	-0.15 – 0.65	0.222	
butterandOil [Yes]	0.02	-0.22 – 0.26	0.850	
Age	0.01	-0.02 – 0.03	0.609	
Sex [Male]	-0.48	-1.05 – 0.08	0.095	
BMI	0.02	-0.06 – 0.09	0.666	

N.B.: Bold fonts indicate statistical significance (p/q value < 0.05).

Table S4. (xi): Linear mixed-effect models of specific gravity-adjusted ETPA as a function of time of urine sampling in the day adjusted for dietary habits and personal care product use (reference: noon).

log(PRPA)

Predictors	Estimates	CI	p	q value
(Intercept)	-2.93	-5.91 – 0.06	0.055	
Time [Morning]	0.57	0.27 – 0.87	**<0.001**	**<0.001**
Time [Night]	0.50	0.21 – 0.78	**0.001**	**0.006**
meat [Yes]	-0.20	-0.51 – 0.11	0.200	
breads [Yes]	-0.22	-0.53 – 0.10	0.184	
beverages [Yes]	0.28	-0.27 – 0.83	0.314	
butterandOil [Yes]	0.22	-0.11 – 0.54	0.198	
Age	0.03	-0.00 – 0.06	0.084	
Sex [Male]	-1.99	-2.83 – -1.15	**<0.001**	**<0.001**
BMI	0.02	-0.09 – 0.13	0.666	

N.B.: Bold fonts indicate statistical significance (p/q value < 0.05).

Table S4. (xii): Linear mixed-effect models of specific gravity-adjusted BUPA as a function of time of urine sampling in the day adjusted for dietary habits and personal care product use (reference: noon).

		log(BUPA)		
Predictors	Estimates	CI	p	q value
(Intercept)	-2.54	-3.97 – -1.11	**0.001**	
Time [Morning]	0.00	-0.21 – 0.22	0.975	
Time [Night]	-0.08	-0.29 – 0.13	0.434	
meat [Yes]	0.06	-0.16 – 0.28	0.604	
breads [Yes]	-0.01	-0.24 – 0.21	0.916	
beverages [Yes]	0.16	-0.22 – 0.54	0.409	
butterandOil [Yes]	-0.11	-0.34 – 0.12	0.330	
Age	0.00	-0.01 – 0.02	0.851	
Sex [Male]	-0.19	-0.58 – 0.20	0.335	
BMI	-0.00	-0.05 – 0.05	0.991	

N.B.: Bold fonts indicate statistical significance (p/q value < 0.05).

Table S4. (xiii): Linear mixed-effect models of specific gravity-adjusted BPA as a function of time of urine sampling in the day adjusted for dietary habits and personal care product use (reference: noon).

		log(BPA)		
Predictors	Estimates	CI	p	q value
(Intercept)	0.39	-0.96 – 1.75	0.569	
Time [Morning]	0.15	-0.07 – 0.38	0.182	
Time [Night]	0.11	-0.11 – 0.33	0.316	
meat [Yes]	-0.08	-0.31 – 0.14	0.462	
breads [Yes]	0.01	-0.22 – 0.24	0.926	
beverages [Yes]	-0.19	-0.57 – 0.19	0.331	

butterandOil [Yes]	0.15	-0.08 – 0.38	0.206
lip products [Yes]	0.33	-0.30 – 0.96	0.301
Age	-0.00	-0.02 – 0.01	0.723
Sex [Male]	0.11	-0.27 – 0.48	0.578
BMI	-0.01	-0.06 – 0.04	0.711

N.B.: Bold fonts indicate statistical significance (p/q value < 0.05).

Table S4. (xiv): Mixed-effect models of specific gravity-adjusted BPS as a function of time of urine sampling in the day adjusted for dietary habits and personal care product use (reference: noon).

		BPS		
Predictors	Odds Ratios	CI	p	q value
(Intercept)	0.72	0.04 – 14.64	0.828	
Time [Morning]	0.41	0.21 – 0.81	**0.010**	**0.047**
Time [Night]	0.89	0.49 – 1.62	0.697	
meat [Yes]	0.64	0.34 – 1.21	0.170	
breads [Yes]	1.10	0.59 – 2.05	0.759	
beverages [Yes]	0.72	0.27 – 1.90	0.503	
butterandOil [Yes]	0.78	0.41 – 1.45	0.428	
Age	0.99	0.96 – 1.02	0.436	
Sex [Male]	0.72	0.33 – 1.59	0.421	
BMI	1.02	0.92 – 1.14	0.706	

N.B.: Bold fonts indicate statistical significance (p/q value < 0.05).

Table S4. (xv): Linear mixed-effect model of specific gravity-adjusted Log(4HNE) as a function of time.

	Log(4HNE)		
Predictors	Estimates	CI	p-value
(Intercept)	3.91	3.42 – 4.40	**<0.001**
Time [Morning]	-0.09	-0.18 – -0.00	**0.041**
Time [Night]	-0.08	-0.17 – 0.01	0.074
Age	0.00	-0.00 – 0.01	0.322
Sex [Male]	0.37	0.23 – 0.51	**<0.001**
BMI	0.00	-0.02 – 0.02	0.979

N.B.: Bold fonts indicate statistical significance (p/q value < 0.05).

Table S5. (i): Linear mixed-effect models for the association between the specific gravity-adjusted log(4HNE) and log(MEP).

	**Log(4HNE)**			
Predictors	Estimates	CI	p	q value
(Intercept)	3.67	3.19 – 4.14	**<0.001**	
MEP [log]	0.09	0.05 – 0.13	**<0.001**	**<0.001**
Age	0.00	-0.00 – 0.01	0.493	
Sex [Male]	0.37	0.23 – 0.50	**<0.001**	**<0.001**
BMI	0.00	-0.01 – 0.02	0.770	

N.B.: Bold fonts indicate statistical significance (p/q value < 0.05).

Table S5. (ii): Linear mixed-effect models for the association between the specific gravity-adjusted log(4HNE) and log(MiBP).

	**Log(4HNE)**			
Predictors	Estimates	CI	p	q value
(Intercept)	3.63	3.16 – 4.11	**<0.001**	
MiBP [log]	0.14	0.09 – 0.20	**<0.001**	**<0.001**
Age	0.00	-0.00 – 0.01	0.478	
Sex [Male]	0.32	0.19 – 0.46	**<0.001**	**<0.001**
BMI	0.00	-0.01 – 0.02	0.749	

N.B.: Bold fonts indicate statistical significance (p/q value < 0.05)

Table S5. (iii): Linear mixed-effect models for the association between the specific gravity-adjusted log(4HNE) and log(MnBP).

	**Log(4HNE)**			
Predictors	Estimates	CI	p	q value
(Intercept)	3.66	3.19 – 4.13	**<0.001**	
MnBP [log]	0.14	0.09 – 0.20	**<0.001**	**<0.001**
Age	0.00	-0.00 – 0.01	0.761	
Sex [Male]	0.33	0.19 – 0.46	**<0.001**	**<0.001**
BMI	0.00	-0.02 – 0.02	0.802	

N.B.: Bold fonts indicate statistical significance (p/q value < 0.05).

Table S5. (iv): Linear mixed-effect models for the association between the specific gravity-adjusted log(4HNE) and log(MBzP).

	**Log(4HNE)**			
Predictors	Estimates	CI	p	q value
(Intercept)	3.83	3.35 – 4.31	**<0.001**	
MBzP [log]	0.11	0.06 – 0.16	**<0.001**	**<0.001**
Age	0.00	-0.00 – 0.01	0.224	
Sex [Male]	0.34	0.20 – 0.48	**<0.001**	**<0.001**
BMI	0.00	-0.02 – 0.02	0.842	

N.B.: Bold fonts indicate statistical significance (p/q value < 0.05).

Table S5. (v): Linear mixed-effect models for the association between the specific gravity-adjusted log(4HNE) and log(SumDEHP).

	Log(4HNE)			
Predictors	Estimates	CI	p	q value
(Intercept)	3.37	2.90 – 3.84	**<0.001**	
SumDEHP [log]	0.18	0.13 – 0.24	**<0.001**	**<0.001**
Age	0.00	-0.00 – 0.01	0.282	
Sex [Male]	0.32	0.19 – 0.45	**<0.001**	**<0.001**
BMI	0.00	-0.02 – 0.02	0.893	

N.B.: Bold fonts indicate statistical significance (p/q value < 0.05).

Table S5. (vi): Linear mixed-effect models for the association between the specific gravity-adjusted log(4HNE) and log(SumDiNP).

	Log(4HNE)			
Predictors	Estimates	CI	p	q value
(Intercept)	3.67	3.22 – 4.13	**<0.001**	
SumDiNP [log]	0.10	0.06 – 0.15	**<0.001**	**<0.001**
Age	0.00	-0.00 – 0.01	0.177	
Sex [Male]	0.33	0.20 – 0.46	**<0.001**	**<0.001**
BMI	-0.00	-0.02 – 0.02	0.873	

N.B.: Bold fonts indicate statistical significance (p/q value < 0.05).

Table S5. (vii): Linear mixed-effect models for the association between the specific gravity-adjusted log(4HNE) and log(SumDINCH).

	**Log(4HNE)**			
Predictors	Estimates	CI	p	q value
(Intercept)	3.84	3.37 – 4.31	**<0.001**	
SumDINCH [log]	0.11	0.07 – 0.16	**<0.001**	**<0.001**
Age	0.00	-0.00 – 0.01	0.261	
Sex [Male]	0.32	0.18 – 0.46	**<0.001**	**<0.001**
BMI	0.00	-0.02 – 0.02	0.766	

N.B.: Bold fonts indicate statistical significance (p/q value < 0.05).

Table S5. (viii): Linear mixed-effect models for the association between the specific gravity-adjusted log(4HNE) and log(Oh.MPHP).

	**Log(4HNE)**			
Predictors	Estimates	CI	p	q value
(Intercept)	4.06	3.58 – 4.53	**<0.001**	
oh MPHP [log]	0.12	0.08 – 0.17	**<0.001**	**<0.001**
Age	0.00	-0.00 – 0.01	0.379	
Sex [Male]	0.34	0.20 – 0.47	**<0.001**	**<0.001**
BMI	0.00	-0.02 – 0.02	0.850	

N.B.: Bold fonts indicate statistical significance (p/q value < 0.05).

Table S5. (ix): Linear mixed-effect models for the association between the specific gravity-adjusted log(4HNE) and log(MEPA).

	Log(4HNE)			
Predictors	Estimates	CI	p	q value
(Intercept)	3.68	3.22 – 4.14	**<0.001**	
MEPA [log]	0.13	0.10 – 0.17	**<0.001**	**<0.001**
Age	-0.00	-0.01 – 0.00	0.685	
Sex [Male]	0.47	0.33 – 0.60	**<0.001**	**<0.001**
BMI	0.00	-0.02 – 0.02	0.982	

N.B.: Bold fonts indicate statistical significance (p/q value < 0.05).

Table S5. (x): Linear mixed-effect models for the association between the specific gravity-adjusted log(4HNE) and log(ETPA).

	Log(4HNE)			
Predictors	Estimates	CI	p	q value
(Intercept)	3.87	3.39 – 4.35	**<0.001**	
ETPA [log]	0.09	0.06 – 0.12	**<0.001**	**<0.001**
Age	0.002	-0.003 – 0.007	0.420	
Sex [Male]	0.42	0.28 – 0.55	**<0.001**	**<0.001**
BMI	-0.001	-0.02 – 0.02	0.903	

N.B.: Bold fonts indicate statistical significance (p/q value < 0.05).

Table S5. (xi): Linear mixed-effect models for the association between the specific gravity-adjusted log(4HNE) and log(PRPA).

	**Log(4HNE)**			
Predictors	Estimates	CI	p	q value
(Intercept)	3.95	3.47 – 4.43	**<0.001**	
PRPA [log]	0.04	0.02 – 0.06	**<0.001**	**<0.001**
Age	0.001	-0.004 – 0.007	0.570	
Sex [Male]	0.45	0.31 – 0.60	**<0.001**	**<0.001**
BMI	-0.001	-0.02 – 0.02	0.936	

N.B.: Bold fonts indicate statistical significance (p/q value < 0.05).

Table S5. (xii): Linear mixed-effect models for the association between the specific gravity-adjusted log(4HNE) and log(BUPA).

	**Log(4HNE)**			
Predictors	Estimates	CI	p	q value
(Intercept)	4.14	3.65 – 4.63	**<0.001**	
BUPA [log]	0.11	0.08– 0.15	**<0.001**	**<0.001**
Age	0.002	-0.003 – 0.008	0.344	
Sex [Male]	0.39	0.26 – 0.53	**<0.001**	**<0.001**
BMI	0.000	-0.02 – 0.02	0.999	

N.B.: Bold fonts indicate statistical significance (p/q value < 0.05).

Table S5. (xiii): Linear mixed-effect models for the association between the specific gravity-adjusted log(4HNE) and log(BPA).

	**Log(4HNE)**			
Predictors	Estimates	CI	p	q value
(Intercept)	3.82	3.34 – 4.26	**<0.001**	
BPA [log]	0.13	0.09 – 0.17	**<0.001**	**<0.001**
Age	0.003	-0.002 – 0.008	0.257	
Sex [Male]	0.36	0.24 – 0.49	**<0.001**	**<0.001**
BMI	0.001	-0.015 – 0.02	0.869	

N.B.: Bold fonts indicate statistical significance (p/q value < 0.05).

Table S5. (xiv): Mixed-effect models for the association between the specific gravity-adjusted log(4HNE) and BPS.

	**Log(4HNE)**			
Predictors	Estimates	CI	p	q value
(Intercept)	3.86	3.38 – 4.35	**<0.001**	
BPS [>LOD]	-0.02	-0.11 – 0.08	0.767	
Age	0.003	-0.003 – 0.008	0.335	
Sex [Male]	0.37	0.23 – 0.51	**<0.001**	**<0.001**
BMI	<0.001	-0.02 – 0.02	0.986	

N.B.: Bold fonts indicate statistical significance (p/q value < 0.05).

Table S6. Comparison of the urinary concentrations of metabolites of phthalates from studies reported on the HBM4EU dashboard.

Sampling period	Country	Biomarkers	N	P25	P50	P75	Sample type (Urine)
2008-2009	Belgium (Adolescents)	MEHHP	209	16.70	23.28	33.6	First morning
		MEOHP		21.33	28.80	45.52	
		MBzP		18.86	33.00	77.60	
		MEHP		2.640	4.08	6.62	
		MnBP		28.40	43.20	67.64	
	Belgium (Adults)	MEHHP	204	12.51	20.33	33.45	
		MEOHP		13.65	24.00	40.29	
		MBzP		14.6	30.00	56.37	
		MEHP		2.00	4.00	7.34	
		MnBP		27.99	45.86	78.10	
2011-2012	Slovenia	MEHHP	120	14.85	21.76	34.88	First morning
		MEOHP		8.04	11.20	18.76	
		MBzP		3.73	6.30	10.01	
		MEHP		2.46	4.58	8.41	
		MEP		29.35	63.51	160.66	
		MiBP		33.14	45.40	71.22	
		MnBP		19.88	31.94	53.47	
2011-2013	Belgium	MEP	150	26.86	58.05	128.06	First morning
2011-2015	Belgium	MEHHP	301	7.94	11.56	17.14	First morning
		MEOHP		5.86	8.51	13.00	
		MBzP		4.48	8.80	16.42	
		MEHP		2.26	3.60	5.94	
		MiBP		41.43	63.24	105.57	
		MnBP		27.92	40.80	59.60	
2012	Norway	MEHHP	56	17.25	22.94	35.18	First morning<br>First morning
		MEHHP		10.85	14.50	25.83	
		MEOHP		6.46	9.09	14.79	
		MBzP		4.43	6.03	9.23	
		MEHP		1.55	2.66	4.69	
		MiBP		31.69	41.17	53.32	
		MnBP		27.16	40.00	56.13	
		OH-MiNP		2.67	3.74	7.36	
		oxo-MiNP		1.33	1.90	4.42	

Table S7. Comparison of the urinary concentrations of metabolites of DINCH from studies reported on the HBM4EU dashboard.

Sampling period	Country	Biomarkers	N	P25	P50	P75	Sample type (Urine)
				Corrected for SG (µg/L)			
2011-2015	Belgium	OH-MINCH	156	0.33	0.52	1.04	First morning
		oxo-MINCH		0.27	0.52	0.95	
				(µg/L)			
2009	Germany	OH-MINCH		<LOD	<LOD	0.13	24hrs
		oxo-MINCH		<LOD	<LOD	<LOD	
2009	Denmark	OH-MINCH	100	0.07	0.72	3.39	Spot urine
2010	Germany	OH-MINCH	60	0.07	0.15	0.5	24hrs

		oxo-MINCH		<LOD	0.07	0.19	
2010-2013	Germany	OH-MINCH	236	0.13	0.32	0.79	24hrs
		oxo-MINCH		0.07	0.18	0.42	
2011-2015	Belgium	OH-MINCH	156	0.02	0.32	0.71	First morning
		oxo-MINCH		0.17	0.31	0.588	
2015-2017	Germany	OH-MINCH	120	0.35	0.6	1.16	24hrs
		oxo-MINCH		0.15	0.29	0.60	
2017	Denmark	OH-MINCH	100	0.72	1.53	3.81	Spot urine

Table S8. Comparison of the urinary concentrations of bisphenols from studies reported on the HBM4EU dashboard.

Sampling period	Country	Biomarkers	N	P25	P50	P75	Sample type (Urine)
2008-2009	Belgium	BPA (total)	196	1.53	2.48	3.83	First morning
	Slovenia	BPA (free)	95	<LOD	<LOD	0.38	First morning
		BPA (total)	107	0.82	2.88	4.64	
	Belgium	BPA (total)	150	1.35	2.15	3.41	First morning
	Norway (Children, 6-19years)	BPA (total)	54	2.67	3.67	4.72	First morning
		BPF (total)	52	<LOD	<LOD	<LOD	
		BPS (total)	52	<LOD	<LOD	<LOD	
	Norway (Mothers)	BPA (total)	56	2.98	5.06	6.21	First morning
		BPF (total)		<LOD	<LOD	<LOD	
		BPS (total)		<LOD	<LOD	<LOD	
	Belgium	BPA (total)	194	1.44	2.1	3.50	Spot urine

Table S9. Comparison of the urinary concentrations of metabolites of parabens in different countries.

Year	Country	N	Biomarkers	P50 (μg/L)
2021	China	319	MeP	5.78
			EtP	0.39
			PrP	0.35
			BuP	0.01
			BzP	0.02
2018	Denmark	195	MeP	11.50
			EtP	1.26
			PrP	2.98
2018	India	41	MeP	6.28
			EtP	0.25
			PrP	0.39
2018	America	4370	MeP	68.4
			EtP	1.10
			PrP	8.30
